# Supplementary material for: Carcinoembryonic antigen-expressing oncolytic measles virus derivative in recurrent glioblastoma: a phase 1 trial
Source: Nat Commun. 2024 Jan 12;15:493. doi: 10.1038/s41467-023-43076-7 (PMC10786937; doi:10.1038/s41467-023-43076-7)
Supplement: Supplementary file 1 — Supplementary Information [file 41467_2023_43076_MOESM1_ESM.pdf]

Carcinoembryonic Antigen-Expressing Oncolytic Measles Virus Derivative in Patients with Recurrent Glioblastoma: a Phase 1 Trial

Supplementary Information

Supplementary Figure 1. Treatment related adverse events by treatment group and dose level. (A) Treatment related adverse events by treatment group. (B) Treatment related adverse events by dose level. Source data are provided as a Source Data file.

1A

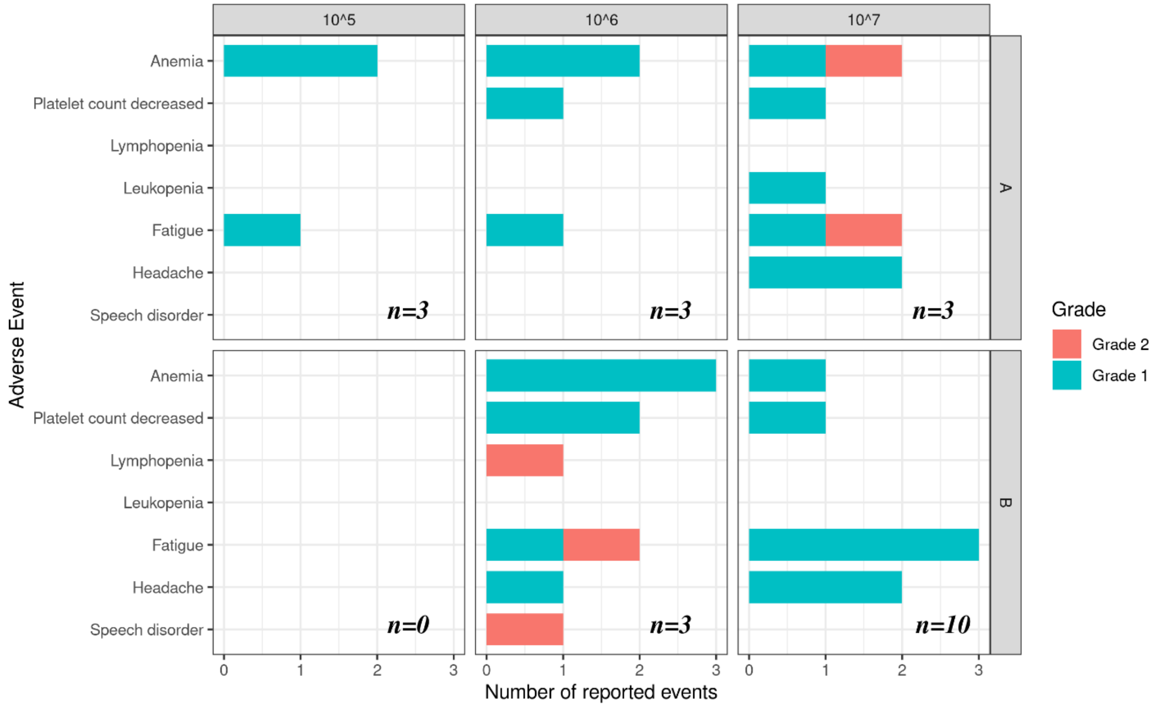

*Group B patients received 2 viral doses for a total dose of $2x10^6$ and $2x10^7$ respectively

1B

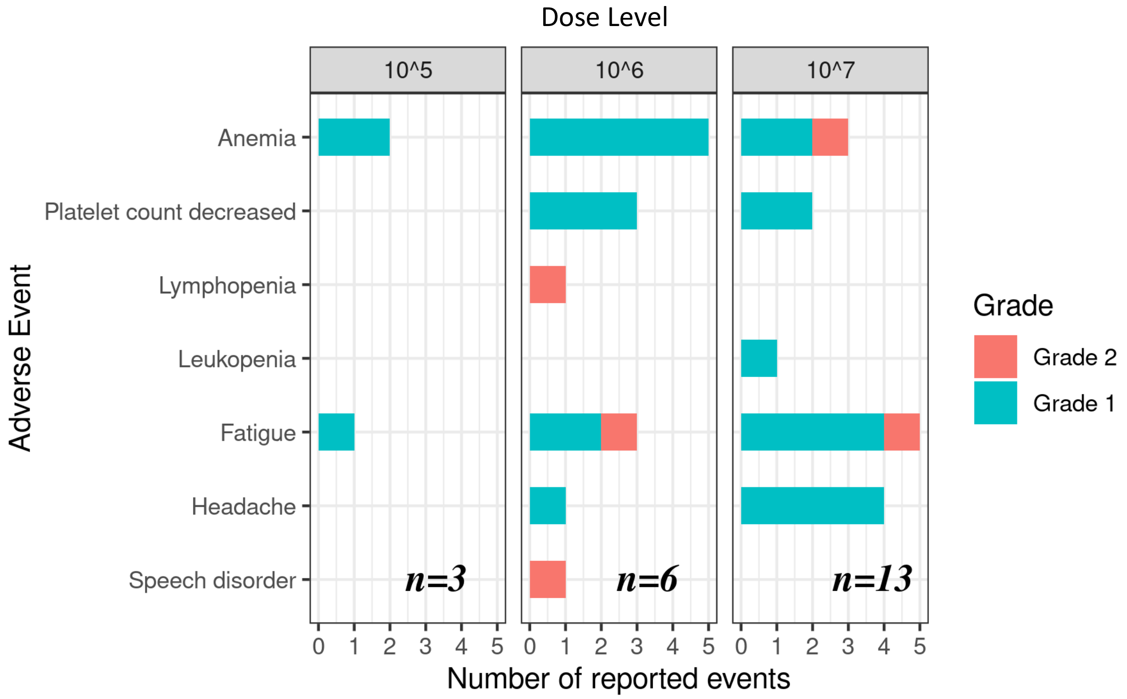

Dose Level
10^5
10^6
10^7
Adverse Event
Anemia
Platelet count decreased
Lymphopenia
Leukopenia
Fatigue
Headache
Speech disorder
n=3
n=6
n=13
0 1 2 3 4 5
Number of reported events
Grade
Grade 2
Grade 1

Supplementary Figure 2. Impact of corticosteroid use at baseline on viral replication and TIL infiltration. (A) Viral genome copies by corticosteroid use at baseline (n=12 Group B patients with available virus replication data, corticosteroid no n=5, yes n=7). Center line indicates median. Box indicates first and third quartiles. Whiskers extend to minimum and maximum values within 1.5*interquartile range; outlying points are plotted separately. Wilcoxon rank sum 2-sided p-values shown. (B) Post treatment change in TIL cells by corticosteroid use at baseline (n=11 Group B patients with available log fold CD4, CD8, CD20 data, corticosteroid no n=4, yes n=7). Source data are provided as a Source Data file.

2A

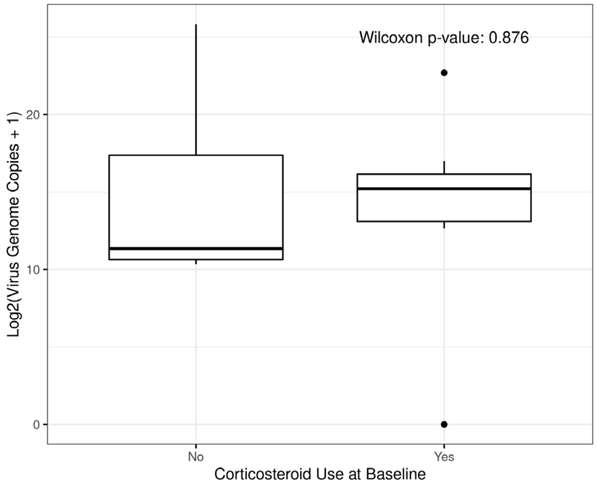

2B

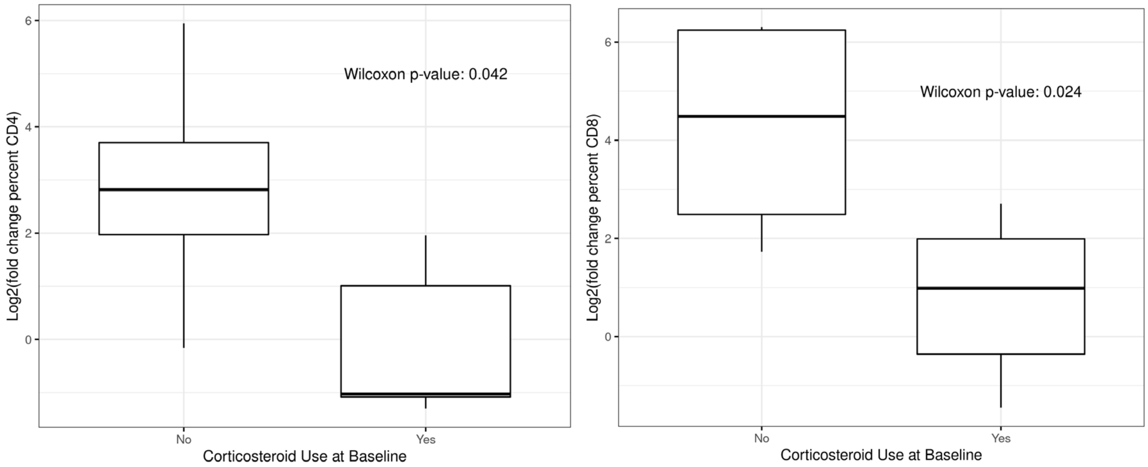

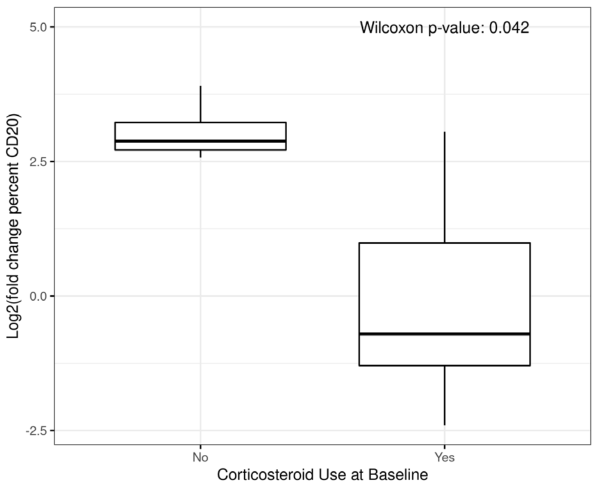

Wilcoxon p-value: 0.042
5.0
2.5
0.0
-2.5
Log2(fold change percent CD20)
No
Yes
Corticosteroid Use at Baseline

Supplementary Figure 3. Pre- versus post-treatment CD4+ T cell infiltration and correlation with DLDA score. (A) Pre- versus post-treatment CD4+ T cell infiltration (Wilcoxon signed-rank test, 2-sided p=0.831, n = 11 Group B patients with paired pre/post-treatment CD4 data). (B) Correlation between DLDA score and CD4+ T cell infiltration post-treatment (Spearman's rho = -0.52, 95% CI: -0.85 to 0.12, 2-sided p=0.107p-value based on asymptotic t approximation, p=0.107, n=11 Group B patients with available pre/post treatment CD4 log2 fold change CD8 data). Source data are provided as a Source Data file.

3A

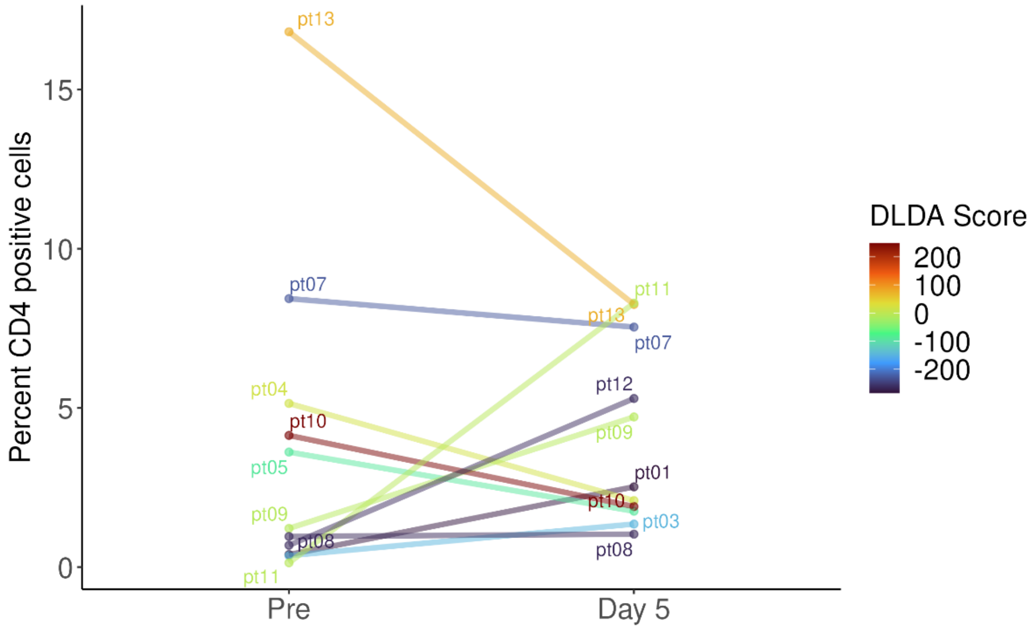

3B

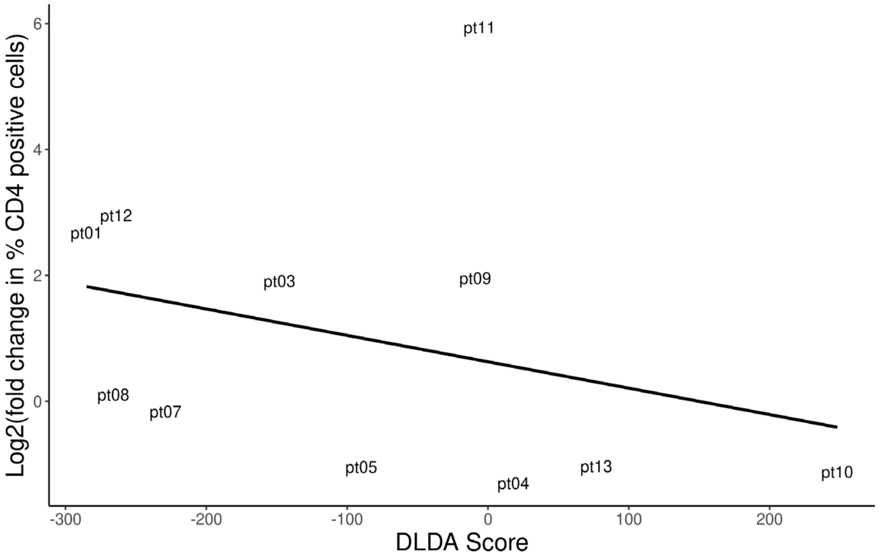

Log2(fold change in % CD4 positive cells)
6
4
2
0
pt11
pt12
pt01
pt03
pt09
pt08
pt07
pt05
pt04
pt13
pt10
-300
-200
-100
0
100
200
DLDA Score

Supplementary Figure 4. PFS and OS outcomes according to tumor IDH status). (A) PFS by IDH mutation status. (B) OS by IDH mutation status. (C) PFS outcomes of all patients vs IDH wild type patients. (D) OS outcomes of all patients vs IDH wild type patients. Source data are provided as a Source Data file.

4A

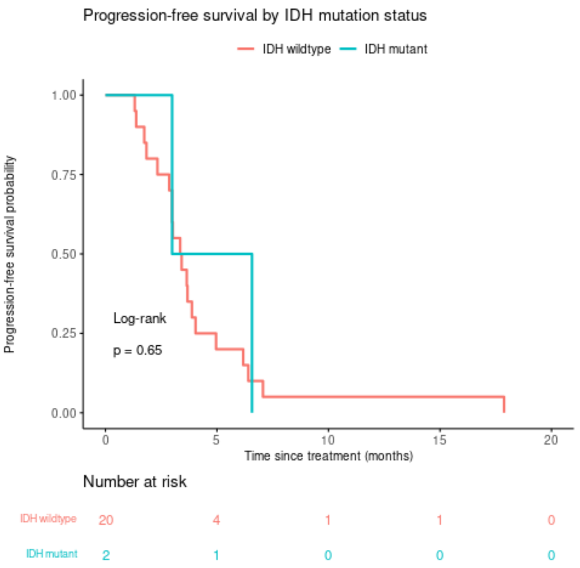

4B

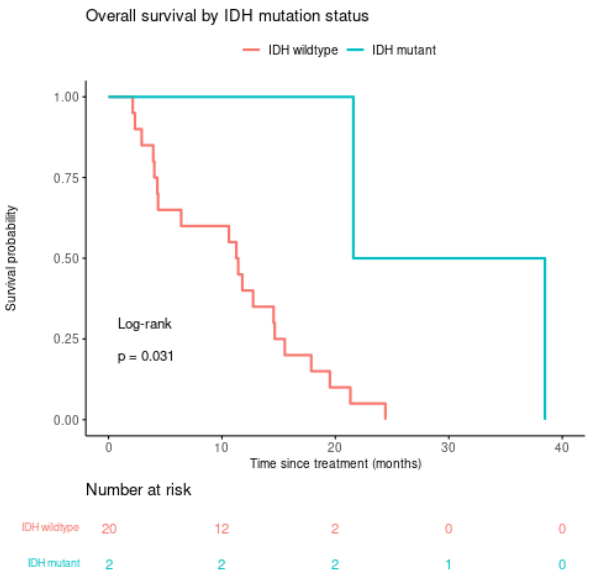

4C

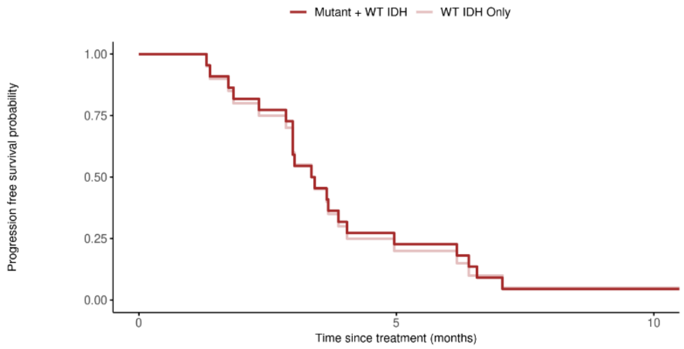

4D

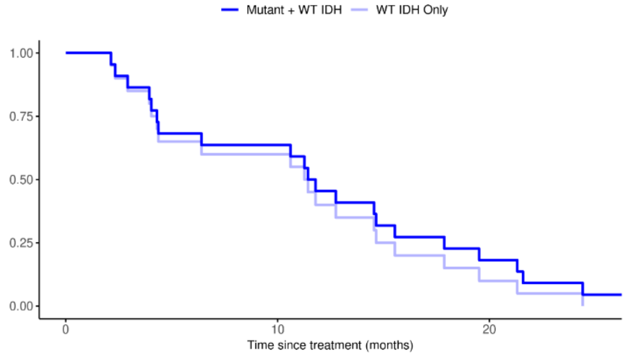

Supplementary Figure 5. MRI imaging evolution following MV-CEA treatment. (A) Post-gadolinium T1-weighted MRI one day prior to the measles virus administration surgery, depicting the recurrent, enhancing, centrally necrotic, left temporal glioblastoma (white arrows). (B) Postoperative MRI after day 1 surgery showing the administration catheter (black arrow) placed within the enhancing tumor. (C) Postoperative MRI after day 5 surgery with second viral dose administered in resection cavity demonstrating resection of the enhancing mass with removal of the catheter (white arrow). (D) MRI 1 month after surgery and two doses of measles virus administration, demonstrating a thin nonspecific enhancing rim around the resection cavity (white arrow). (E) MRI 3.5 months following resection and measles virus treatments, showing shrinkage of the resection cavity but progression of nonspecific solid enhancement around it (white arrows). (F) MRI 5 months after measles virus treatment, showing further progression of solid enhancing tissue (white arrows), which prompted the initiation of bevacizumab. Corresponding T2-FLAIR images are shown in (I-N). Images (O) and P) show relative cerebral blood volume (rCBV) from perfusion imaging in color scale, registered and superimposed on post-gadolinium T1-weighted imaging, at the 3.5 and 5 month time points after day 5 resection. In O), only a small portion of the enhancing lesion had questionably increased rCBV (black arrow), while most of the enhancing lesion had low blood volume suggesting that the increase in enhancement was due to inflammation and not true tumor progression. Minimal change in T2 FLAIR at the same timepoint, as shown in M), further supports lack of progression. In P) the inferior aspect of the lesion developed increased rCVB indicating progression at 5 months following the last viral dose.

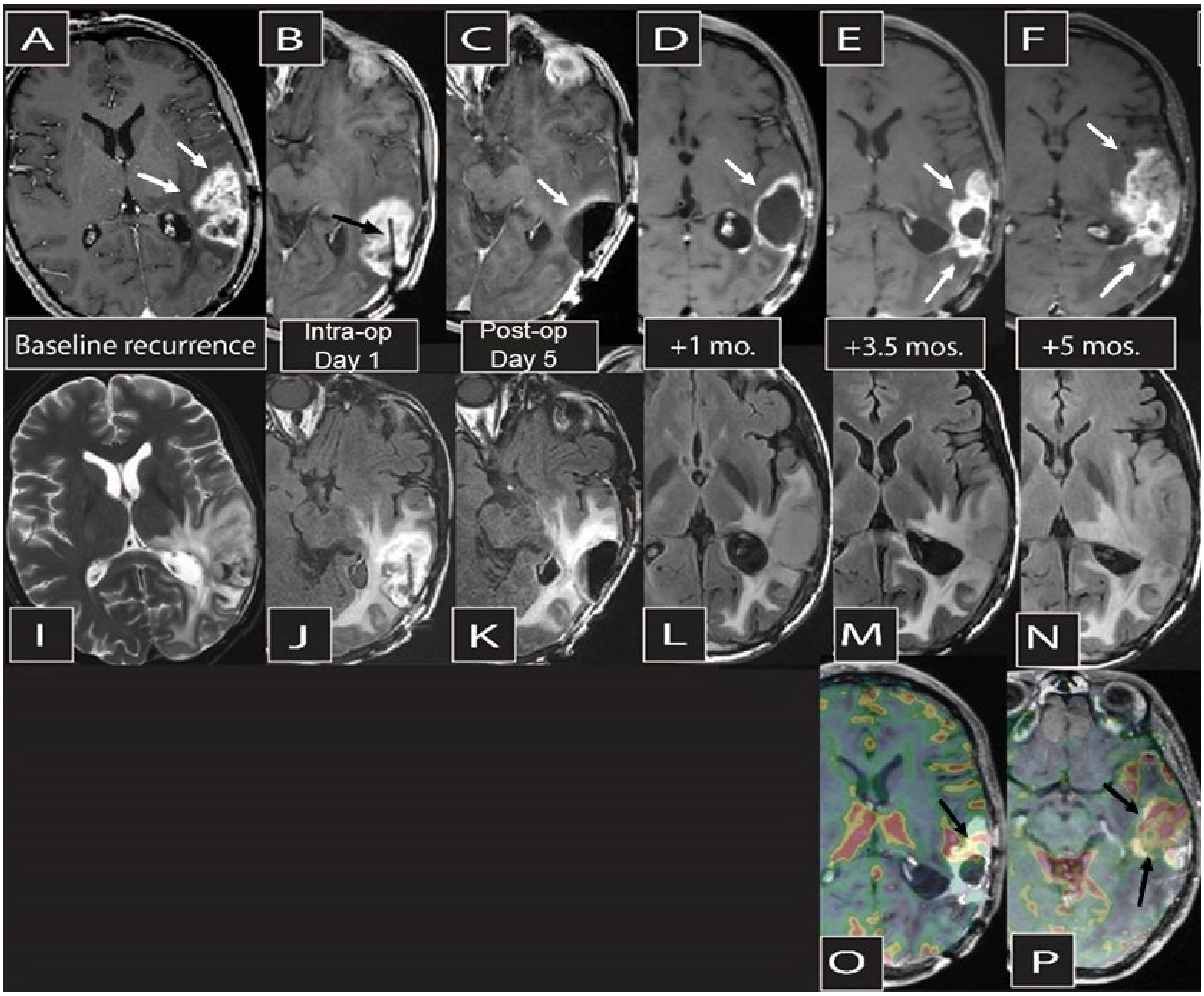

Supplementary Figure 6. Trial schema

Group A	Day 1
	En block tumor resection with confirmation of diagnosis + Injection of MV-CEA in resection cavity

- Viral Doses: 10^5 – 10^7 TCID50

Group B	Day 1	Day 5
	Tumor Biopsy + Stereotactic injection of MV-CEA	En block tumor resection + Injection of MV-CEA in resection cavity

- Viral Doses: 2 $x10^6$ – $2x10^7$ TCID50

Supplementary Figure 7. Tumor resection specimens on day 5 following Day 1 **administration of MV-CEA via intratumoral catheter.** Following Day 1 intratumoral administration of MV-CEA in Group B patients, the catheter was left in place; on day 5, tumor and catheter were resected en block. Representative examples from two study patients are shown.

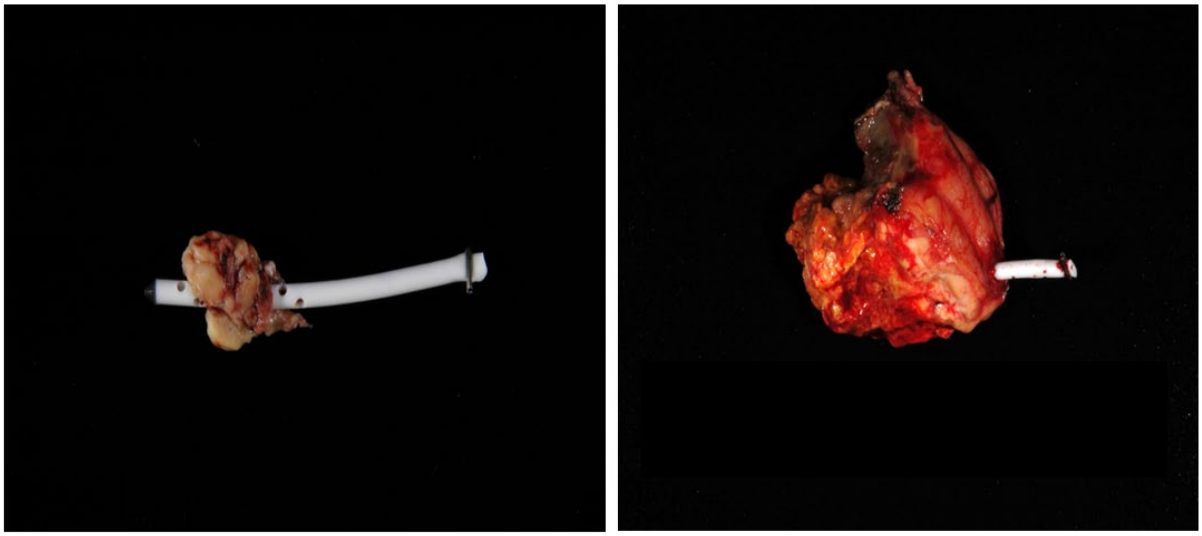

Supplementary Figure 8. Schematic representation of correlative laboratory analysis

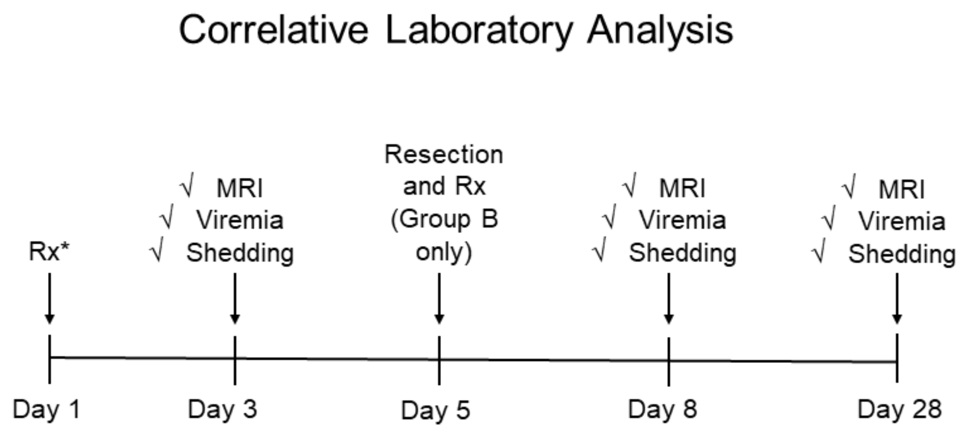

- CEA kinetics: Pre-Rx at 6 hours, days 2, 3, 4, 5, 8 and 28
- Additional analysis: Anti MV-Abs, immunoglobulin, CD4 and CD8 counts, complement: baseline and on day 28
- *Rx – viral treatment

Supplementary Figure 9. Pre vs post treatment CEA levels by treatment group. Post-treatment CEA measurements were taken a median of 29 days after treatment day 1 (range of 18-35 days). Mean change in CEA levels from pre- to post-treatment overall was 0.127 ng/ml (95% CI: -0.093, 0.348, paired t-test two-sided p=0.244, n=22 patients). The mean change in CEA levels for Group A was 0.089 ng/ml (95% CI: -0.256, 0.434, paired t-test two-sided p=0.569, n=9 patients); for Group B, 0.154 ng/ml (95% CI: -0.176, 0.484, paired t-test two-sided p=0.329, n=13 patients). Source data are provided as a Source Data file.

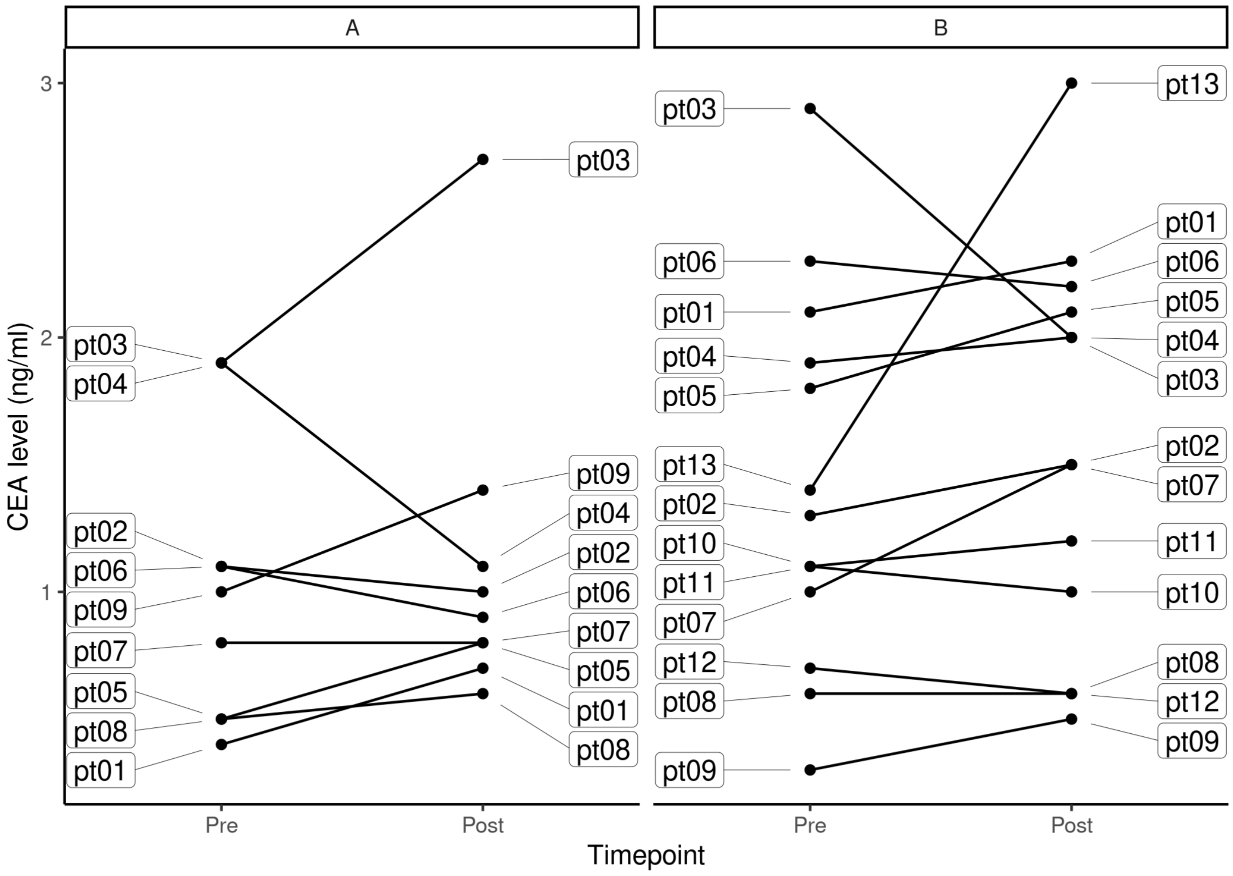

Supplementary Figure 10. **DLDA score groupings (low < -250, intermediate -250 to 150, high > 150) correlation with viral replication.** (A) Pearson's correlation coefficient (r) with 95% CI and two-sided p-value based on a t-distribution with n-2 degrees of freedom (n=12 patients with available virus replication data). (B) Summary of viral genome copies by DLDA group. Source data are provided as a Source Data file.

10A

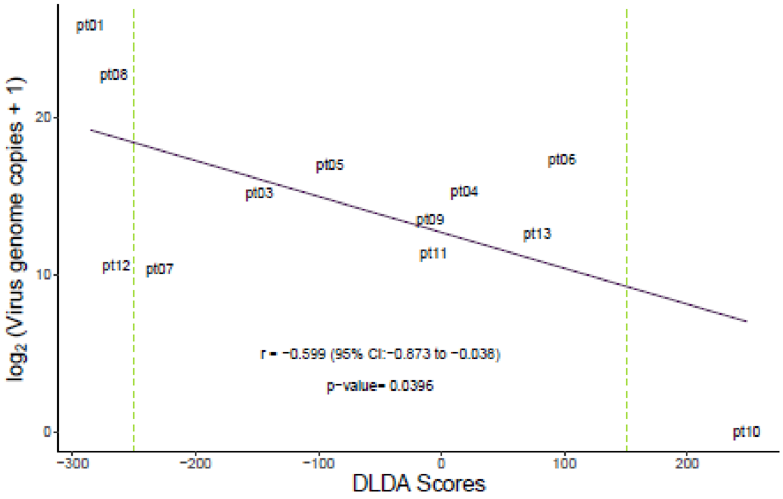

10B

		Viral genome copies				
DLDA score group	N	Mean	Median	SD	Min	Max
High (>=150)	1	0	0		0	0
Intermediate (-250 < x < 100)	8	5.02x10^4	2.50x10^4	6.44x10^4	1.30x10^3	1.70x10^5
Low (<= -250)	3	2.23x10^7	6.80x10^6	32.85x10^6	1.60x10^3	6.00x10^7

Supplementary Figure 11. Viral replication vs. CD46, SLAM, and Nectin-4 levels. (A) CD46, (B) Nectin 4, (C) SLAM. Spearman's Rho and two-sided p-value from an asymptotic t approximation (n=12 patients with available viral replication data). Source data are provided as a Source Data file.

11A

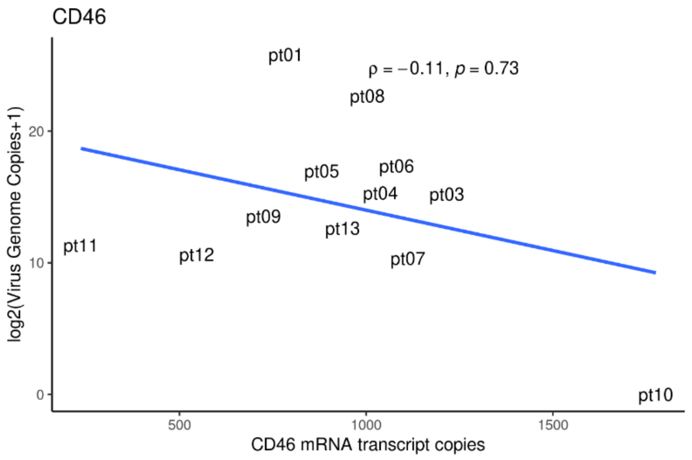

11B

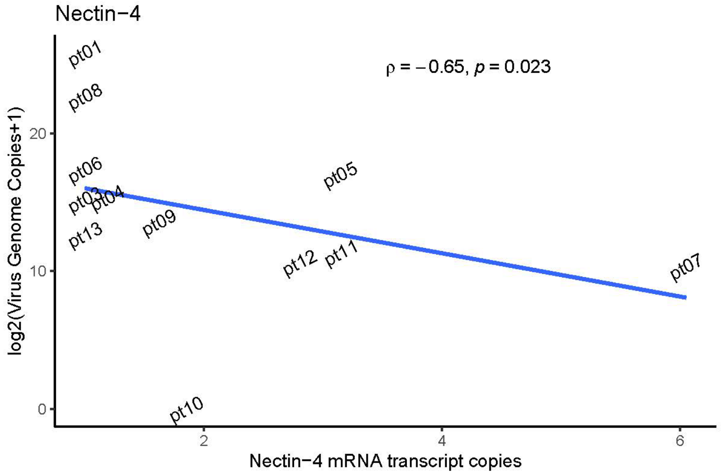

11C

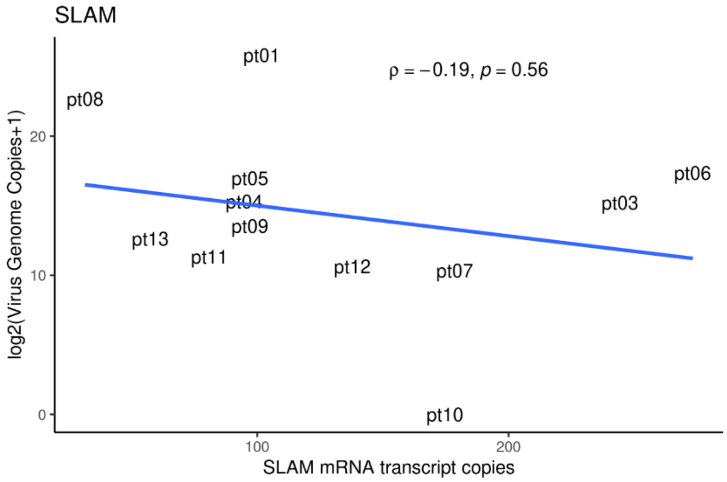

Supplementary Table 1. IDH mutation status, MGMT status, and tumor grade

Group A

Patient Number	Glioma Grade	IDH Mutation Status	MGMT Status
pt01	4	negative	negative
pt02	4	positive	NA
pt03	4	negative	negative
pt04	4	negative	negative
pt05	4	positive	negative
pt06	4	negative	positive
pt07	4	negative	indeterminate
pt08	4	negative	negative
Pt09	4	negative	negative

NA = not available

Group B

Patient Number	Glioma Grade	IDH Mutation Status	MGMT Status
pt01	4	negative	negative
pt02	4	negative	NA
pt03	4	negative	negative
pt04	3	negative	negative
pt05	4	negative	negative
pt06	4	negative	negative
pt07	4	negative	negative
pt08	4	negative	indeterminate
pt09	4	negative	negative
pt10	4	negative	negative
pt11	4	negative	negative
pt12	4	negative	negative
pt13	4	negative	negative

NA = not available

Supplementary Table 2. Treatment-related adverse events by treatment Group and grade

	**Group A: 9 evaluable patients**		**Group B: 13 evaluable patients**	
	Adverse event grade*			
	1-Mild	2-Mod	1-Mild	2-Mod
**Adverse event n (% of evaluable patients by group)**				
Anemia	5 (55.6%)	1 (11.1%)	4 (30.8%)	0
Fatigue	3 (33.3%)	1 (11.1%)	4 (30.8%)	1 (7.7%)
Headache	2 (22.2%)	0	3 (23.1%)	0
Platelet count decreased	2 (22.2%)	0	3 (23.1%)	0
Leukocyte count decreased	1 (11.1%)	0	0	0
Lymphocyte count decreased	0	0	0	1 (7.7%)
Speech disorder	0	0	0	1 (7.7%)

*Max AE grade by patient

Supplementary Table 3. Input and output viral genome copies/ µg RNA in representative study patients

Patient Number	Pretreatment tumor volume (cm3)	Dose level units	Genome copies per dose	Viral Genome copies/µg RNA (Input Day 1)	Viral Genome copies/µg RNA (Day 5)
1	21.1	1×10^6	2.3×10^8	5.45×10^3	6×10^7
6	12.1	1×10^7	3.88×10^9	1.6×10^5	1.7×10^5
8	14.5	1×10^7	3.88×10^9	1.34×10^5	6.8×10^6
9	27.5	1×10^7	3.88×10^9	7.05×10^4	1.2×10^4
11	3.1	1×10^7	3.88×10^9	6.26×10^5	2.6×10^3

Supplementary Table 4. OS by continuous DLDA score (per 50-unit increase)

Characteristic	HR[1]	95% CI[1]	p-value[2]
DLDA score	0.93	0.77, 1.11	0.43

[1]HR = Hazard Ratio, CI = Confidence Interval

[2]Wald test

Supplementary Table 5. OS by SLAM (10-unit increase), Nectin-4 (1-unit increase), and CD46 (100-unit increase) expression

Model*	HR[1]	95% CI[1]	p-value[2]
SLAM	1.02	0.94, 1.10	0.63
Nectin-4	1.01	0.72, 1.41	0.96
CD46	0.93	0.76, 1.13	0.46

[1]HR = Hazard Ratio, CI = Confidence Interval

[2]Wald test

*Results are from 3 separate univariable models

Supplementary Table 6. Unrelated AEs

6A Unrelated AEs, overall and by Group

	Overall	Group A	Group B
Number of evaluable patients	22	9	13
Adverse event n (% of evaluable patients)			
Seizure	8 (36.4%)	3 (33.3%)	5 (38.5%)
Hemoglobin decreased	7 (31.8%)	0	7 (53.8%)
Speech disorder	7 (31.8%)	3 (33.3%)	4 (30.8%)
Ataxia	6 (27.3%)	4 (44.4%)	2 (15.4%)
Fatigue	6 (27.3%)	1 (11.1%)	5 (38.5%)
Peripheral motor neuropathy	6 (27.3%)	5 (55.6%)	1 (7.7%)
Headache	4 (18.2%)	2 (22.2%)	2 (15.4%)
Confusion	3 (13.6%)	2 (22.2%)	1 (7.7%)
Lymphocyte count decreased	3 (13.6%)	0	3 (23.1%)
Platelet count decreased	3 (13.6%)	0	3 (23.1%)
Peripheral sensory neuropathy	2 (9.1%)	1 (11.1%)	1 (7.7%)
Pyramidal tract syndrome	2 (9.1%)	0	2 (15.4%)
Thrombosis	2 (9.1%)	1 (11.1%)	1 (7.7%)
Anxiety	1 (4.5%)	1 (11.1%)	0
Colonic obstruction	1 (4.5%)	1 (11.1%)	0
Depressed level of consciousness	1 (4.5%)	0	1 (7.7%)
Dyspnea	1 (4.5%)	1 (11.1%)	0
Hyperglycemia	1 (4.5%)	1 (11.1%)	0
Leukocyte count decreased	1 (4.5%)	0	1 (7.7%)
Localized edema	1 (4.5%)	0	1 (7.7%)
Neutrophil count decreased	1 (4.5%)	0	1 (7.7%)
Pneumonia(gr 3/4 ANC)	1 (4.5%)	1 (11.1%)	0

6B Unrelated AEs, overall and by grade (maximum grade per patient per event)

	Overall	Grade 1	Grade 2	Grade 3
Adverse event n (% of 22 evaluable patients)				
Seizure	8 (36.4%)	0	6 (27.3%)	2 (9.1%)
Hemoglobin decreased	7 (31.8%)	6 (27.3%)	1 (4.5%)	0
Speech disorder	7 (31.8%)	0	6 (27.3%)	1 (4.5%)
Ataxia	6 (27.3%)	2 (9.1%)	4 (18.2%)	0
Fatigue	6 (27.3%)	5 (22.7%)	1 (4.5%)	0
Peripheral motor neuropathy	6 (27.3%)	3 (13.6%)	2 (9.1%)	1 (4.5%)
Headache	4 (18.2%)	3 (13.6%)	1 (4.5%)	0
Confusion	3 (13.6%)	2 (9.1%)	0	1 (4.5%)
Lymphocyte count decreased	3 (13.6%)	0	1 (4.5%)	2 (9.1%)
Platelet count decreased	3 (13.6%)	3 (13.6%)	0	0
Peripheral sensory neuropathy	2 (9.1%)	1 (4.5%)	1 (4.5%)	0
Pyramidal tract syndrome	2 (9.1%)	0	1 (4.5%)	1 (4.5%)
Thrombosis	2 (9.1%)	0	0	2 (9.1%)
Anxiety	1 (4.5%)	0	1 (4.5%)	0
Colonic obstruction	1 (4.5%)	0	1 (4.5%)	0
Depressed level of consciousness	1 (4.5%)	0	0	1 (4.5%)
Dyspnea	1 (4.5%)	0	1 (4.5%)	0
Hyperglycemia	1 (4.5%)	0	0	1 (4.5%)
Leukocyte count decreased	1 (4.5%)	0	1 (4.5%)	0
Localized edema	1 (4.5%)	0	1 (4.5%)	0
Neutrophil count decreased	1 (4.5%)	0	1 (4.5%)	0
Pneumonia(gr 3/4 ANC)	1 (4.5%)	0	0	1 (4.5%)

Supplementary Table 7. Treatment-related AEs by treatment Group and dose level

		Group A Dose Level			Group B Dose Level	
	Overall	10^5	10^6	10^7	2 x 10^6	2 x 10^7
Number of evaluable patients	22	3	3	3	3	10
Adverse event n (% of evaluable patients)						
Hemoglobin decreased	10 (45.5%)	2 (66.7%)	2 (66.7%)	2 (66.7%)	3 (100%)	1 (10%)
Fatigue	9 (40.9%)	1 (33.3%)	1 (33.3%)	2 (66.7%)	2 (66.7%)	3 (30%)
Headache	5 (22.7%)	0	0	2 (66.7%)	1 (33.3%)	2 (20%)
Platelet count decreased	5 (22.7%)	0	1 (33.3%)	1 (33.3%)	2 (66.7%)	1 (10%)
Leukocyte count decreased	1 (4.5%)	0	0	1 (33.3%)	0	0
Lymphocyte count decreased	1 (4.5%)	0	0	0	1 (33.3%)	0
Speech disorder	1 (4.5%)	0	0	0	1 (33.3%)	0

Supplementary Note. Protocol

Immediately following this page.

Mayo Clinic Cancer Center

MC0671: Phase I Trial of a Measles Virus Derivative Producing CEA (MV-CEA) in Patients with Recurrent Glioblastoma Multiforme (GBM)

Study Chair: Evanthia Galanis, M.D.*
Mayo Clinic
200 First Street, SW
Rochester, MN 55905
507/284–2511
507/284-1803 (FAX)

Study Co-Chairs: Jan C. Buckner, M.D.
Brian P. O'Neill, M.D.
Fredric B. Meyer, M.D.
John L.D. Atkinson, M.D.
Richard W. Marsh, M.D.
David G. Piepgras, M.D.
Caterina Giannini, M.D.√
Timothy Kaufmann, M.D.
Stephen J. Russell, M.D., Ph.D.√
Mark J. Federspiel, Ph.D.√
Gregory A. Poland, M.D.
Roberto Cattaneo, Ph.D. √
Ian Parney, M.D.

Statistician: S Keith Anderson, M.S. √

* Investigator having NCI responsibility for this protocol.
√ Study contributor(s) not responsible for patient care.

Protocol Resources

Questions:	Contact Name:
Drug administration, infusion pumps, nursing guidelines	Lisa A Kottschade, RN, MSN, CNP<br>Nurse Resource<br>Phone: Phone/voicemail (507) 293-0571<br>E-mail: Kottschade.lisa@mayo.edu
Protocol document, consent form, Regulatory issues	See Protocol Catalog for current RPS assignment:<br>http://ccswww:8686/catalog/catalog/styDetail.jsf?sty_node=4221
Adverse Events (AdEERS, MedWatch, AML/MDS)	Patricia G McNamara<br>Mayo Cancer Center SAE Coordinator<br>Phone: (507) 266-3028<br>E-mail: mcnamara.patricia@mayo.edu
Laboratory Contact for Viremia and Virus Shedding	Paula J Zollman<br>Location: Guggenheim 1802<br>Phone: (507) 266-0386<br>E-mail: zollman.paula@mayo.edu
Study Coordinator	Susan (Sue) M Steinmetz<br>Phone: 507/266-5280<br>Fax: 507/284-2251<br>E-mail: steinmetz.susan@mayo.edu
Forms completion, eligibility	Sharon F Solinger<br>Phone: (507) 266-1714<br>Fax: (507) 266-7240<br>E-mail: Solinger.Sharon@mayo.edu

Table of Contents

MC0671: Phase I Trial of a Measles Virus Derivative Producing CEA (MV-CEA) in Patients with Recurrent Glioblastoma Multiforme (GBM) 1

Protocol Resources 2

Table of Contents 3

Schema 4

1.0 Background 5

2.0 Goals 9

3.0 Patient Eligibility 9

4.0 Test Schedule 12

5.0 Grouping Factor 18

6.0 Registration/Randomization Procedures 18

7.0 Protocol Treatment 19

8.0 Dose Modification 22

9.0 Ancillary Treatment 23

10.0 Adverse Event (AE) Reporting and Monitoring 24

11.0 Treatment Evaluation 27

12.0 Descriptive Factor 28

13.0 Treatment/Follow–up Decision at Evaluation of Patient 29

14.0 Pharmacologic/Correlative 30

15.0 Drug Information 32

16.0 Statistical Considerations and Methodology 33

17.0 Pathology Considerations 35

18.0 Data Collection Procedure 36

19.0 Budget Considerations 36

20.0 References 38

Schema

Prior to discussing protocol entry with the patient, call the Randomization Center (507-284-2753) for Treatment Arm and dose level and to ensure that a place on the protocol is open to the patient.

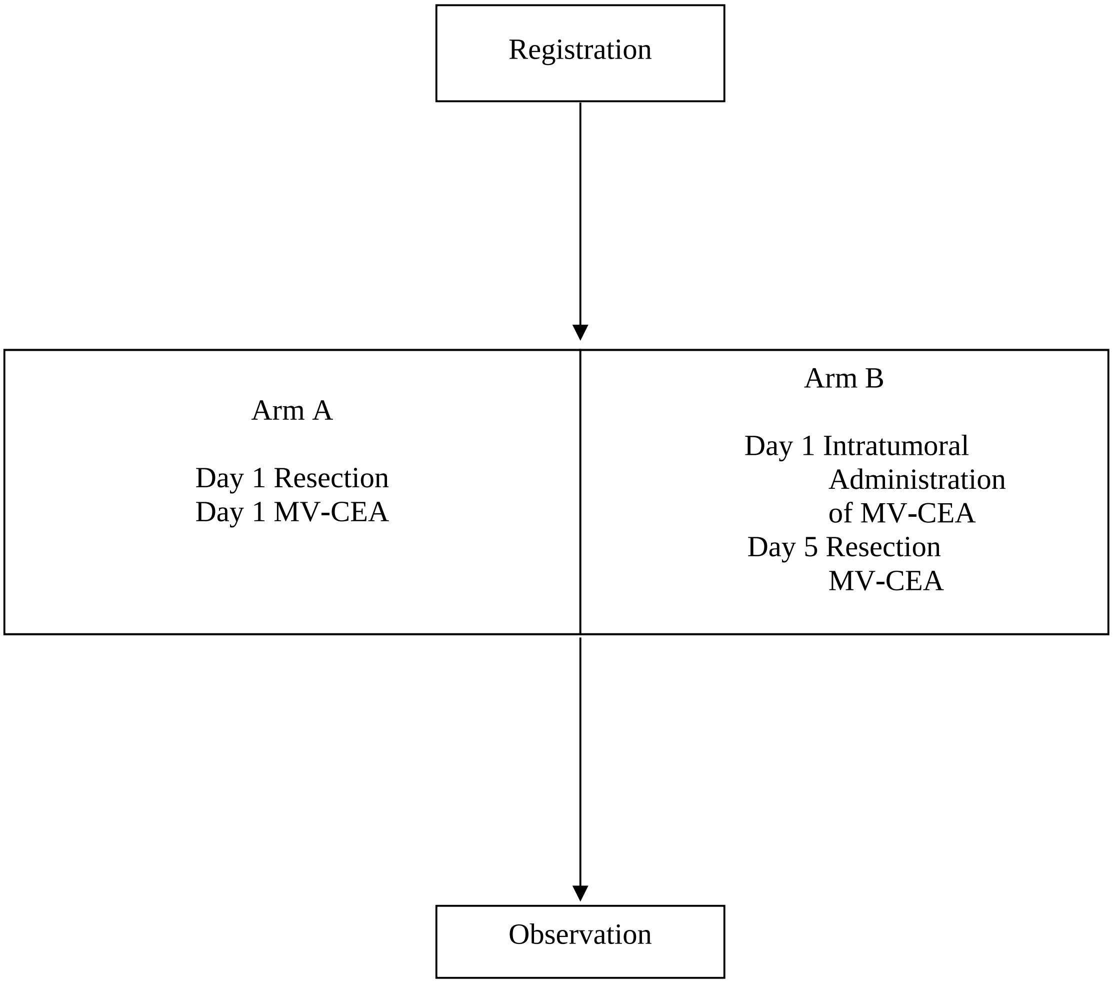

*Patients will be accrued Arm A until MTD is determined for Arm A. Subsequent patients will then be accrued to Arm B. The treatment arms will NOT be open to accrual simultaneously.

1.0 Background

1.1 **Glioblastoma multiforme:** Glioblastoma multiforme is the most frequent primary brain tumor in adults and accounts for the majority of 18,500 primary brain tumor cases diagnosed each year in the United States [Jemal, 2005]. It represents one of the most lethal malignancies with a median survival of 12-15 months despite multimodality treatment. Gliomas represent a promising target for gene transfer approaches given their limited ability to metastasize, but despite promising preclinical data [Ram et al, 1997;Shand et al, 1999;Berger et al, 1997;Oldfield et al, 1993; Rainov, 2000], significant clinical benefit has not been materialized to date. Replicating viruses tested in clinical trials for recurrent gliomas include herpes viruses [Markert et al, 2000;Rampling et al, 2000] the conditionally replicating adenovirus ONYX-015 [Chiocca et al, 2004] and reovirus. In phase I clinical trials of HSV strains and of the conditionally replicating adenovirus ONYX-015 in recurrent glioma patients, no dose limiting toxicity has been encountered. *In vivo* replication of these viruses in the patients could not be consistently evaluated, however, pointing to inherent difficulties, when testing viral therapies in brain tumor patients [Markert et al, 2000], where assessment of viral replication requires repeat brain biopsies, an invasive and potentially risky procedure. In all completed trials in recurrent glioma patients, the lack of convincing evidence of clinically significant antitumor activity points towards the need to further improve the existing viral systems or develop novel, more potent viral agents. They also emphasize the need to devise systems that could allow monitoring of viral propagation in the tumor in order to accomplish dose optimization and individualization of treatment.

1.2 **Measles Virus as a cancer therapy:** Measles virus is a negative strand, RNA virus, whose genome includes six protein products [Griffin et al, 1996]. Three of these proteins participate in the formation of the viral envelope; the H-protein is the surface glycoprotein which mediates measles virus attachment to its receptors, the CD46 molecule [Dorig et al, 1993], and the SLAM receptor (the latter being predominantly present on activated B and T cells). The F-protein is responsible for cell fusion after viral attachment has taken place. During natural infection with the measles virus, the virus replicates in susceptible tissues causing a very characteristic cytopathic effect, with development of multinucleated giant cells (syncytia). Cells infected by measles virus express F and H-proteins on their membranes and therefore become highly fusogenic. These cells can cause fusion not only with other infected cells but also with uninfected neighboring cells. Although wild type measles virus can lead to a potentially serious infectious disease, attenuated strains (vaccine strains) of measles virus have an excellent safety record and have resulted in significant decreases in measles incidence and mortality worldwide [Cutts et al, 1994].

1.3 Oncolytic measles virus strains have potent antitumor activity against gliomas: Our group has developed a novel antitumor approach to treat glioblastoma multiforme. Based on the observation that a combination of two of the measles virus membrane glycoproteins, the F and H fusogenic membrane glycoproteins had potent antitumor activity against glioma cell lines and xenografts, we hypothesized that replicating attenuated vaccine strain of measles virus (MV) the natural carrier of these proteins, could amplify this antitumor potential with an excellent safety profile. In contrast to normal cells, tumor cells, including glioma cells, overexpress the measles virus receptor CD46 which allows for preferential tumor cell targeting [Phuong L et al, 2003; Anderson et al, 2004]. In order to develop a method that would allow non-invasive real-time monitoring of viral replication in the tumor and dose optimization, we created trackable viruses that express the soluble marker CEA.

The MV-CEA virus was constructed by introducing the extracellular N-terminal domain of

human CEA in the measles virus backbone derived from the Edmonston vaccine lineage [Peng et al, Nat Med, 2002]. CEA is a 677 amino acid glycoprotein that crosses the blood brain barrier and can be detected in the serum, as demonstrated in cases of colon cancer, a CEA producing malignancy, when it metastasizes to the brain. CEA is not normally expressed in gliomas and therefore, measurable levels of CEA peptide in the serum after viral treatment would allow us to monitor in vivo the spread and/or elimination of the virus and measure the profile of viral gene expression over time.

Infection of a variety of glioblastoma cell lines, both established and primary cell lines derived from Mayo Clinic glioblastoma patients and maintained as subcutaneous xenografts, showed significant cytopathic effect consisting of cell to cell fusion and extensive syncytia formation, leading to apoptotic cell death [Phuong L et al, 2003]. The antitumor effect of MV-CEA was assessed in established subcutaneous U87 glioma xenografts in BALB/c nude mice. Animals with established subcutaneous U87 xenografts treated with MV-CEA (total dose of 8 x 107 TCID50) had significant tumor regression and prolongation of survival as compared to control mice ($p<0.001$ and $p=0.007$, respectively). Furthermore, treatment of intracranial U87 xenografts with MV-CEA at a total dose of 1.8 x 106 TCID50 led to statistically significant tumor regression and prolongation of survival as compared to control animals ($p=0.0028$ and $p=0.02$, respectively), with 60% of the animals remaining alive and tumor free at 80 days from tumor inoculation [Phuong et al, 2003]. Significant antitumor efficacy of measles virus strains was also observed against a variety of other animal glioma models, including orthotopic glioblastoma models deriving from glioblastoma primary lines, such as GBM39 ($p=0.0079$) and GBM14 ($p=0.037$) [Petell and Galanis, unpublished data].

In all preclinical efficacy studies, the kinetic profile of viral gene expression in mice could easily be followed by determining serum levels of the virally encoded marker peptide (CEA). CEA correlated well with tumor response and the significant antitumor efficacy of the engineered virus was not compromised [Phuong et al, 2003].

1.4 Preclinical assessment of toxicity in Rhesus macaques and in a transgenic mouse model of measles virus pathogenecity: Attenuated vaccine strains of measles virus of the Edmonston vaccine lineage from where our viruses also derived have an excellent safety record with millions of individuals having been safely vaccinated [Cutts et al, 1994]. Nevertheless, in our trial we exploit a different route of administration and the proposed dose escalation reaches doses that are higher than what normally is used for measles vaccination (103 to 104 TCID50). In consultation with the FDA, the preclinical assessment of toxicity was performed in non-human primates, Rhesus macaques and Ifnar CD46ko transgenic mice. Rhesus macaques (Macacca Mullata) are naturally susceptible to measles virus infection and have been extensively used to study measles virus pathogenesis. CNS inoculation of measles virus vaccine strains is the standard method by which the measles virus vaccine lots are tested for neurotoxicity. Nevertheless, the cost and complexity of studies in primates significantly hampers our ability to perform such studies in large numbers of animals. Therefore, identifying an appropriate smaller animal model represented a key element in the design of our toxicology studies.

Rodents do not normally express the measles virus receptor CD46 and SLAM, therefore, they cannot be used to assess toxicity of oncolytic measles virus strains. Dr. R. Cattaneo from the Mayo Clinic Molecular Medicine Program has developed a very appealing transgenic mouse model of measles virus pathogenecity by inserting a yeast artificial chromosome containing the human CD46 receptor gene and knocking out the INF-α-β receptor gene. These Ifnarko CD46 Ge transgenic mice express human CD46 in a tissue distribution that replicates human

CD46 expression and are susceptible to MV Edmonston infection. CD46 distribution in the brain of CD46 transgenic mice has been shown to mimic the distribution in humans [Mrkic B et al, 1998]. Furthermore, studies of intracerebral inoculation of Ifnarko CD46 GE mice with MV Edmonston have demonstrated that this can be suitable animal model for neurotoxicity assessment [Mrkic B et al, 1998].

1.5 **Evaluation of toxicity after CNS administration of MV-CEA in Rhesus macaques:**Five adult measles immune Rhesus macaques were used in this toxicology study. Immune animals were chosen in order to mimic the study population in the proposed clinical trial. Two macaques received 106 TCID50 on days 1 and 5 to mimic the repeat dose administration schedule in our trial, two received 105 TCID50 on days 1 and 5, and one animal received vehicle control on the same days. The injections were administered in the frontal lobe of the animals, a common location for glioblastoma multiforme using previously established stereotactic coordinates [Driesse et al, 1998]. By direct brain weight conversion the highest dose was 1.5 x higher than maximum human dose in the proposed trial and 3 x higher the maximum dose that the normal brain could be exposed in our study. Animals were monitored closely for clinical signs including rash, fever, conjunctivitis, and neurologic symptoms such as level of consciousness, motor function, gait, activity level, behavior, posture and facial symmetry. No evidence of neurological or other toxicity has been observed to date, at 9 months from viral administration. Hematological/biochemical parameters were normal at all time points. No CEA elevation was detected pointing to absence of virus propagation. There was no evidence of viremia in PBMC by QRT-PCR, except for a single time point in one of the high dose animals, and no shedding in buccal mucosa secretions was observed. In addition to lack of clinical signs, there was no laboratory evidence of encephalitis on CSF exam performed on day 9 after administration of the first viral dose. No virus was detected in the CSF by QRT-PCR or Vero cell overlay. MRI imaging at 4 months revealed no imaging abnormalities. These results support the safety of administration of MV-CEA into primary CNS tumors.

1.6 Toxicity after CNS administration of MV-CEA in Ifnarko CD46 Ge mice: The $Ifnar^{ko}$ CD46 Ge mouse model is a very sensitive animal model of measles virus neurotoxicity. Although human patients are not lacking the interferon-α-β receptor gene, nevertheless, in consultation with FDA we applied this model in order to assess the neurotoxicity of MV-CEA in immune mice, thus mimicking the worst case scenario in our patient population. One hundred and twenty, 6 to 8 weeks old measles immune Ifnarko CD46 Ge mice were assigned to three dose groups, one group receiving vehicle control, the second receiving 104 TCID50 on days 1 and 5, and the third receiving 105 TCID50 on days 1 and 5. By direct brain weight conversion, this is approximately equivalent to 35 x the maximum human dose in our trial. General appearance, activity level, body weight and key hematological/biochemical parameters were monitored in all groups of animals, without evidence of abnormality. In order to assess toxicity, 30 animals from each group were euthanized on early (days 4 and 8), and intermediate (day 26) time points and tissues from multiple organs were subjected to histological analysis. Close clinical monitoring revealed no signs of clinical toxicity or neurotoxicity. Histologic examination of
ten vital organs—brain, heart, lung, liver, pancreas, kidneys, spleen, ovary, testes, peritoneum, and skeletal muscles revealed no significant difference between vehicle treated animals and MV-CEA treated animals. No CEA elevation was observed. An additional group of animals is due to be euthanized in January 2006 (day 90), in order to assess late toxicity/persistence.

1.7 **Safety data from a phase I trial of intraperitoneal administration of MV-CEA in patients with ovarian cancer:**MV-CEA is currently undergoing phase I testing in ovarian cancer patients. Twelve patients have been treated to date in doses ranging from 10^3 to 10^6 TCID50. In

this phase I trial, MV-CEA is administered in the peritoneal cavity through a peritoneal port-a-cath. Repeat administration of the virus for up to six cycles is allowed, if good treatment tolerance and no disease progression. Intraperitoneal administration of MV-CEA has been well tolerated so far with no dose limiting toxicity having been observed. Furthermore, there was no evidence of shedding in urine or oral secretions and no evidence of immunosuppression. No significant increase in the titers of anti-measles virus antibody in serum or peritoneal fluid after repeat administration has been observed. No patient has developed anti-CEA antibodies.

Viral genomes were detected transiently in the peripheral blood mononuclear cells of two patients. Preliminary evidence of biologic activity has been observed with 3 patients having > 50% reduction in the levels of tumor marker CA-125, and 5 patients having stable disease lasting from 2 to 9 months as their best objective response.

1.8 Summary of rationale for study design One of the great challenges in clinical applications of gene transfer has been our limited ability to monitor viral propagation and transfer of the genetic material of interest to the target tissue and, therefore, individualize the treatment in order to achieve optimal safety and efficacy. Thus, we have created a recombinant, attenuated Edmonston B vaccine strain of measles virus, MV-CEA, that has been genetically engineered to express an inert soluble marker peptide (human carcinoembryonic antigen, CEA), and has potent antitumor activity against glioma lines and xenografts. The construction of this virus variant (which retains its antitumor effect) allows us to monitor the activity of the virus by testing CEA levels in the peripheral blood.

Thus, MV-CEA represents a unique new therapeutic agent for glioblastoma multiforme. We have shown that viral propagation correlates with CEA levels. Based in these data, we propose to proceed with a phase I trial of intratumoral and resection cavity administration of the Edmonston's strain measles virus, genetically engineered to produce CEA in patients with recurrent gliomas.

2.0 Goals

2.1 To assess the safety and toxicity of intratumoral and resection cavity administration of an Edmonston's strain measles virus genetically engineered to produce CEA (MV-CEA) in patients with recurrent glioblastoma multiforme

2.2 To determine the maximum tolerated dose (MTD) of MV-CEA.

2.3 To characterize viral gene expression at each dose level as manifested by CEA titers.

2.4 To assess viremia, viral replication, and measles virus shedding/persistence following intratumoral administration.

2.5 To assess humoral and cellular immune response to the injected virus.

2.6 To assess in a preliminary fashion antitumor efficacy of this approach.

3.0 Patient Eligibility

Prior to discussing protocol entry with the patient, call the Randomization Center (507-284-2753) for dose level and to ensure that a place on the protocol is open to the patient.

3.1 Treatment

3.11 Required Characteristics

3.111 ≥18 years.

3.112 **Recurrent Grade 3 or 4 glioma, including astrocytoma, oligodendroglioma or mixed glioma with histologic confirmation at initial diagnosis or recurrence.**

3.113 Candidate for gross total or subtotal resection.

3.114 Adequate hematologic, renal and hepatic function tests as indicated by the following laboratory values obtained ≤14 days prior to registration:

- ANC ≥1500/µL
- PLT ≥100,000/µL
- Total bilirubin ≤1.5 x upper normal limit (ULN)
- AST ≤2 x ULN
- Creatinine ≤2.0 x ULN
- Hgb ≥9.0 gm/dL
- PT and aPTT ≤1.3 x ULN

3.115 Ability to provide informed consent.

3.116 ECOG performance status (PS) 0, 1 or 2.

3.117 Anti-measles virus immunity as demonstrated by IgG anti-measles antibody levels of ≥ 1.1 EU/ml as determined by Enzyme Immunoassay

3.118 Normal serum CEA levels (<3 ng/ml) at the time of registration.

3.119a Willing to provide biologic specimens as required by the protocol.

3.119b Negative serum pregnancy test done ≤7 days prior to registration (for women of childbearing potential only).

3.2 Contraindications

3.21 Any of the following:
- Pregnant women
- Nursing women
- Men or women of childbearing potential who are unwilling to employ adequate contraception

3.22 Active infection ≤5 days prior to registration.

3.23 History of tuberculosis or history of PPD positivity.

3.24 Any of the following therapies:
- Chemotherapy ≤ 4 weeks prior to registration (6 wks for nitrosourea-based chemotherapy)
- Immunotherapy ≤ 4 weeks prior to registration
- Biologic therapy ≤ 4 weeks prior to registration
- Bevacizumab ≤ 12 weeks prior to registration
- Non-cytotoxic antitumor drugs, i.e., small molecule cell cycle inhibitors ≤ 2 weeks prior to registration
- Radiation therapy ≤ 6 weeks prior to registration
- Any viral or gene therapy prior to registration.

3.25 Failure to fully recover from acute, reversible effects of prior chemotherapy regardless of interval since last treatment.

3.26 New York Heart Association classification III or IV.

3.27 Requiring blood product support

3.28 Inadequate seizure control.

3.29a Expected communication between ventricles and resection cavity as a result of surgery.

3.29b HIV-positive test result, or history of other immunodeficiency.

3.29c History of organ transplantation.

3.29d History of chronic hepatitis B or C.

3.29e Other concurrent chemotherapy, immunotherapy, radiotherapy or any ancillary therapy considered investigational (utilized for a non-FDA-approved indication and in the context of a research investigation).

3.29f Exposure to household contacts ≤15 months old or household contact with known immunodeficiency.

3.29g Allergy to measles vaccine or history of severe reaction to prior measles vaccination.

4.0 Test Schedule

4.1 Treatment Arm A

4.11 COHORT 1 (First 3 evaluable patients on Dose Level 1 only)

		CYCLE 1				OBSERVATION[14]			
**Tests and Procedures**	**≤14 days prior to registration**	**Day 1**	**Day 3 ±1 day**	**7 ±2 days after resection**	**21 ±2 days after resection**	**7 weeks after tumor resection ±5 days**	**q 2 mo until prog**	**3 mo after prog[13]**	**12 mo after prog[13] and yearly[10] thereafter[14]**
History and exam, neurologic examination[1], wt, ECOG PS, vital signs (BP, pulse, temp), assessment of toxicity	X[1]		X[1]	X[1]	X[1]	X[1]	X[1]	X[1]	X[1]
Height	X								
Hematology group WBC, ANC, Lymphocyte count, Hgb, PLT	X		X	X	X	X	X	X	X
PT, APTT	X		X						
Chemistry group (Na, K, Ca, creatinine, AST, alkaline phosphatase, bilirubin total and direct, total protein, albumin)	X		X	X	X	X	X	X	X
HIV blood test[R]	X								
Serum pregnancy[2]	X								
Tumor Measurement / Evaluation of indicator lesion (MRI or CT[4, 11])	X		X[3]		X	X	X	X	X[12]
CEA[5,R]	X		X	X	X	X	X	X	X
Assessment of immune competence[R] (Immunoglobulins, CD4 & CD8 counts, complement levels)	X				X				
Measles virus immunity[R] Serum anti-measles IgG	X			X	X		X		
LPA[6], IFN-γ ELISPOT	X				X				
Anti-CEA antibodies[R]	X			X	X				
Assessment of viral shedding[7,R]	X		X	X	X				
Assessment of Viremia (PBMC)[8,R]	X		X	X	X[8,9]	X[8]	X[8]	X[8]	X[8]
Tumor resection		X							

Footnotes continue on next page

1. Neurologic examination to be performed by neurologist.
2. For women of childbearing potential only. Must be done ≤7 days prior to registration.
3. Day 3 MRI can be performed earlier at the surgeon's discretion if clinically indicated.
4. Only if MRI contraindicated
5. CEA will also be determined in addition at 6 hrs after viral administration and on Days 2, 3, and 5.
6. LPA=Lymphoproliferative Assay, IFN-γ ELISPOT; evaluates cellular immunity against measles virus. It will be performed at the laboratory of Dr Greg Poland.
7. Viral shedding will be assessed by RT-PCR of throat gargle specimens and urine samples prior to treatment, and Days 14 and 28 after tumor resection. These will be done in the CRU unless patient is hospitalized.
8. Testing for viremia will be discontinued after day 28 if two consecutive negative tests are obtained.
9. If viremia is present on Day 28 repeat testing weekly until negative.
10. Up to 15 years as per gene therapy trial guidelines.
11. MRI perfusion sequences to be included.
12. Tumor measurements not required after the 12 month post-progression follow-up.
13. Patients should be encouraged to return to Mayo Clinic Rochester for this evaluation, but if unable to return, evaluation can be performed by the local physician.
14. Assessments during Observation may be performed by the local physician, provided no evidence of viremia remains and follow-up imaging will be provided to study team at Mayo Clinic in Rochester, MN.

R Research funded.

4.12 NON-COHORT 1 PATIENTS ON ARM A

		Cycle 1				Observation[14]		
**Tests and Procedures**	**≤14 days prior to registration**	**Day 1**	**Day 3 ±1 day**	**14 days after tumor resection ±3 days**	**28 days after tumor resection ±3 days**	**q 2 mo until prog**	**3 mo after prog[13]**	**12 mo after prog[13] and yearly[10] thereafter[14]**
History and exam, neurologic examination[1], wt, ECOG PS, vital signs (BP, pulse, temp), assessment of toxicity	X[1]		X[1]	X[1]	X[1]	X[1]	X[1]	X[1]
Height	X							
Hematology group WBC, ANC, Lymphocyte count, Hgb, PLT	X		X	X	X	X	X	X
PT APTT	X		X					
Chemistry group (Na, K, Ca, creatinine, AST, alkaline phosphatase, bilirubin total and direct, total protein, albumin)	X		X	X	X	X	X	X
HIV blood test[R]	X							
Serum pregnancy[2]	X							
Tumor Measurement / Evaluation of indicator lesion (MRI or CT[4, 11])	X		X[3]		X	X	X	X[12]
CEA[5,R]	X		X	X	X	X	X	X
Assessment of Immune competence[R] (Immunoglobulins, CD4 & CD8 counts, complement levels)	X				X			
Measles virus immunity[R] Serum anti-measles IgG	X				X	X		
LPA[6], IFN-γ ELISPOT	X				X			
Anti-CEA antibodies[R]	X				X	X		
Assessment of viral shedding[7,R]	X		X	X	X			
Assessment of Viremia (PBMC)[8,R]	X		X	X	X[8,9]	X[8]	X[8]	X[8]
Tumor resection		X						

1. Neurologic examination to be performed by neurologist.
2. For women of childbearing potential only. Must be done ≤7 days prior to registration.
3. Day 3 MRI can be performed earlier at the surgeon's discretion if clinically indicated.
4. Only if MRI contraindicated
5. CEA will also be determined in addition at 6 hrs after viral administration and on days 2, 3, and 5.

6. LPA=Lymphoproliferative Assay, IFN-γ ELISPOT; evaluates cellular immunity against measles virus. It will be performed at the laboratory of Dr Greg Poland.
7. Viral shedding will be assessed by RT-PCR of throat gargle specimens and urine samples prior to treatment, and days 3, 14 and 28 after tumor resection. These will be done in the CRU unless patient is hospitalized.
8. Testing for viremia will be discontinued after day 28 if two consecutive negative tests are obtained.
9. If viremia is present on day 28 repeat testing weekly until negative.
10. Up to 15 years as per gene therapy trial guidelines.
11. MRI perfusion sequences to be included.
12. Tumor measurements not required after the 12 month post-progression follow-up.
13. Patients should be encouraged to return to Mayo Clinic Rochester for this evaluation, but if unable to return, evaluation can be performed by the local physician.
14. Assessments during observation may be performed by the local physician, provided no evidence of viremia remains and follow-up imaging will be provided to study team at Mayo Clinic in Rochester, MN.

R Research funded.

4.2 Treatment Arm B

		Cycle 1							Observation[14]		
**Tests and Procedures**	**≤14 days prior to registration**	**Day 1**	**Day 3**	**Day 4**	**Day 5**	**Day 8 ±1 day**	**14 days after tumor resection ±3 days**	**28 days after tumor resection ±3 days**	**q 2 mo until prog**	**3 mo after prog[13]**	**12 mo after prog[13] and yearly[10] thereafter[14]**
History and exam, neurologic examination[1], wt, ECOG PS, vital signs (BP, pulse, temp), assessment of toxicity	X[1]		X[1]		X	X[1]	X[1]	X[1]	X[1]	X[1]	X[1]
Height	X										
Hematology group WBC, ANC, Lymphocyte count, Hgb, PLT	X		X		X	X	X	X	X	X	X
PT, APTT	X		X			X					
Chemistry group (Na, K, Ca, creatinine, AST, alkaline phosphatase, bilirubin total and direct, total protein, albumin)	X		X		X	X	X	X	X	X	X
HIV blood test	X										
Serum pregnancy test[2,R]	X										
Tumor Measurement /Evaluation of indicator lesion (MRI or CT[4, 11])	X			X[R]		X[3]		X	X	X	X[12]
CEA[5,R]	X		X	X	X	X	X	X	X	X	X
Assessment of Immune competence[R] (Immunoglobulins, CD4 & CD8 counts, complement levels)	X							X			
Measles virus immunity[R] Serum anti-measles IgG	X							X	X		
LPA[6], IFN-γ ELISPOT	X							X			
Anti-CEA antibodies[R]	X							X	X		
Assessment of viral shedding[7,R]	X		X		X	X	X	X			
Assessment of Viremia (PBMC)[8,9,]	X		X		X	X	X	X[8,9]	X[8]	X[8]	X[8]
Intratumoral administration of virus [R]		X									

		Cycle 1							Observation[14]		
**Tests and Procedures**	**≤14 days prior to registration**	**Day 1**	**Day 3**	**Day 4**	**Day 5**	**Day 8 ±1 day**	**14 days after tumor resection ±3 days**	**28 days after tumor resection ±3 days**	**q 2 mo until prog**	**3 mo after prog[13]**	**12 mo after prog[13] and yearly[10] thereafter[14]**
CT or MRI imaging of catheter placement [R,15]		X									
Tumor resection with viral administration					X						

1. Neurologic examination to be performed by neurologist. NOTE: Weight and ECOG PS are not needed on days 3, 5, and 8)
2. For women of childbearing potential only. Must be done ≤7 days prior to registration.
3. Day 8 MRI can be performed earlier at the surgeon's discretion if clinically indicated.
4. Only if MRI contraindicated.
5. CEA will also be determined in addition at 6 hrs after viral administration and daily Days 2-9.
6. LPA=Lymphoproliferative Assay, IFN-γ ELISPOT; evaluates cellular immunity against measles virus. It will be performed at the laboratory of Dr Greg Poland.
7. Viral shedding will be assessed by RT-PCR of throat gargle specimens and urine samples prior to treatment and Days 3, 5, 8, and on Days 14 and 28 after tumor resection. These will be done in the CRU unless patient is hospitalized
8. Testing for viremia will be discontinued after day 28 if two consecutive negative tests are obtained.
9. If viremia is present on Day 28, repeat testing weekly until negative.
10. Up to 15 years as per gene therapy trial guidelines.
11. MRI perfusion sequences to be included.
12. Tumor measurements not required after the 12 month post-progression follow-up.
13. Patients should be encouraged to return to Mayo Clinic Rochester for this evaluation, but if unable to return, evaluation can be performed by the local physician.
14. Assessments during Observation may be performed by the local physician, provided no evidence of viremia remains and follow-up imaging will be provided to study team at Mayo Clinic in Rochester, MN.
15. If indicated

R. Research funded.

5.0 Grouping Factor

Treatment Group: Arm A vs Arm B

6.0 Registration/Randomization Procedures

6.1 Prior to discussing protocol entry with the patient, call the Randomization Center (507-284-2753) for dose level and to ensure that a place on the protocol is open to the patient.

6.2 To register a patient, call (4-2753) or fax (4-0885) a completed eligibility checklist to the Randomization Center between 8 a.m. and 5 p.m. central time, Monday through Friday.

6.3 A signed HHS 310 form must be on file in the Randomization Center before an investigator may register any patients.

6.4 At the time of registration, Randomization Center personnel will verify the following:

- IRB approval
- Patient eligibility
- Existence of a signed consent form
- Existence of a signed authorization for use and disclosure of protected health information.

In addition, the following will be recorded:

- Patient has/has not given permission to store samples for future research of cancer;
- Patient has/has not given permission to store samples for further research or learn about, prevent, or treat other health problems
- Patient has/has not given Mayo permission to give their samples to outside researchers.

6.5 Treatment on this protocol must be administered at Mayo Clinic Rochester under the supervision of a medical oncologist and neurosurgeon.

6.6 Treatment cannot begin prior to registration and must begin ≤14 days after registration.

6.7 Pretreatment tests must be completed within the guidelines specified on the test schedule.

6.8 All required baseline symptoms (see Section 10.3) must be documented and graded in the medical record.

6.9 Patients will be admitted to the CRU for outpatient visits to complete the throat gargle and urine collection for the viral shedding. The clinical research associate (CRA) should inquire with the CRU on availability prior to registering the patient.

7.0 Protocol Treatment

7.1 Treatment Schedule

Treatment Arm A After confirmation of diagnosis, a tumor resection will be performed. The designated viral dose will be diluted in 1 mL of NS and administered in the resection cavity using a 20-gauge blunt tip needle injected 1 to 2 cm in the brain parenchyma in multiple (10) injection sites.

Dose Level	Day 1
As assigned by MCCC Randomization Center	En block tumor resection with confirmation of diagnosis + Injection of MV-CEA in resection cavity

Agent	Dose Level	Dose	Route	Day
MV-CEA	0	10^4 TCID50	RC	1
“	1*	10^5 TCID50	RC	1
“	1.5**	$3*10^5$ TCID50	RC	1
“	2	10^6 TCID50	RC	1
“	3	10^7 TCID50	RC	1

*starting dose level
** only if 1/6 DLTs are observed at dose level 1
RC= Administration in resection cavity

If any DLT is observed at dose level 1 and the total number of DLTs in this dose level is <2/6, dose will be escalated to the intermediate dose level of 3 x 10^5 TCID50.

Treatment Arm B
A silastic ventricular catheter will be placed using stereotactic equipment (Stealth, Compass, or Leksell systems). CT or MRI imaging will be employed to secure catheter placement, if indicated and the designated dose of MV-CEA will be injected into the tumor as a single bolus via the catheter. Based on experience from prior gene transfer studies with intratumoral administration of viral agents, we plan to dilute the virus in 1 mL of normal saline [Lang et al, 2003]. The agent will be administered within a 10-minute period at 0.1 mL per minute, followed by infusion of 0.2 mL of Normal Saline.

The catheter will be left in place to mark the injection site, it will be secured to the dura, and the incision will be closed. The procedure has been previously employed in viral

CNS trials with excellent tolerance [Lang et al, 2003]. The patients will have an open craniotomy on day 5, timing corresponding to the expected maximum viral replication [Phuong et al, 2003]. At that time an en block tumor resection will be performed by circumferentially dissecting the tumor using computer-assisted stereotactic technique, making sure not to dislodge the catheter. After resection, the same dose of virus diluted in 1 mL of NS, will be administered circumferentially around the tumor bed, using a 20-

gauge blunt tip needle injected 1 to 2 cm into the brain parenchyma in multiple (10) injection sites, in an equally spaced pattern. Brain regions defined as potentially functioning based on stereotactic guidance will not be injected.

Dose Level	Day 1	Day 5
As assigned by MCCC Randomization Center	Intratumoral administration of MV-CEA	En block tumor resection + Injection of MV-CEA in resection cavity

Agent	Dose Level	Dose	Route	Day
MV-CEA	-1	10^4 TCID50*	IT/RC	1 and 5
“	0	10^5 TCID50*	IT/RC	1 and 5
“	1*	10^6 TCID50*	IT/RC	1 and 5
“	2	10^7 TCID50*	IT/RC	1 and 5

*starting dose level **if** MTD for Arm A is 10^7 TCID50 IT/RC= Intratumoral administration (day 1) and administration in resection cavity (day 5)

Accrual in Arm B will start after the MTD in Arm A has been established. The MTD from Arm A will be used to determine the starting dose for Arm B; Arm B starting dose will be one dose level less than the MTD found in Arm A (e.g., if the MTD for Arm A is 10^7 TCID50 then starting dose for Arm B will be 10^6 TCID50 days 1 and 5)

Note: As of 10/20/2009 the MTD for Arm A has been established as 10^7 TCID50. Therefore the starting dose level for Arm B is dose level 1, (10^6 TCID50).

7.2 For this protocol, dose-limiting toxicity (DLT) will be defined as an adverse event attributed (definitely, probably, or possibly) to the study treatment <u>and</u> meeting the following criteria:

**Toxicity**	**Definition**
Hematologic	≥grade 3 as per NCI Common Terminology Criteria for Adverse Events v3.0 except grade 3 ANC lasting <72 hours.
Nonhematologic	≥grade 3 as per NCI Common Terminology Criteria v3.0*
Neurologic toxicity	≥grade 2 as per NCI Common Terminology Criteria v3.0
Allergic reaction	The grade 2 allergic reactions asymptomatic bronchospasm and/or urticaria, and ≥grade 3 allergic reactions
Viremia**	Lasting for ≥6 weeks from last viral administration

* ≥Grade 3 nausea, vomiting, or diarrhea will be considered dose-limiting only if patient is receiving the maximum supportive care regimen described in the protocol. Alopecia will not be considered dose limiting.

**Viremia is defined as detection of any titer of the virus by RT-PCR in patient's PBMCs. Detection limit in this assay is 1000 genome copies/μg RNA.

7.3 Both arms will use a cohort of 3 design to determine the MTD of both schedules. The MTD from Arm A will be used to determine the starting dose Arm B. Cohorts of 3 patients will be accrued at a dose level, treated and assessed for toxicity 2 weeks after treatment (see exception below for dose level 1, Arm A). A minimum of three and maximum of 6 patients will be accrued to each dose level per cohort of 3 design characteristics.

7.31 The first three evaluable patients accrued to dose level 1 on Arm A will be enrolled sequentially. Each patient will be treated and followed for three weeks to assess for toxicity before the next patient is enrolled and treated.

7.4 MTD is defined as the dose level below the lowest dose that induces dose-limiting toxicity in at least one-third of patients (at least 2 of a maximum of 6 new patients).

7.41 Three patients will be treated at a given dose level and observed for at least 14 days post-resection to assess toxicity.

7.42 If dose-limiting toxicity (DLT) is not seen in any of the first 3 patients, 3 new patients will be accrued and treated at the next higher dose level. If DLT is seen in 2 or 3 of 3 patients treated at a given dose level, then the next 3 patients will be treated at the next lower dose level.

7.43 If DLT is seen in 1 of 3 patients treated at a given dose level, up to 3 additional patients will be enrolled and treated at the same dose level. If DLT is seen in at least one of these additional three patients (≥2 of 6), the MTD will have been exceeded and further accrual will cease to this cohort. If dose-limiting toxicity (DLT) is not seen in any of the three additional patients, 3 new patients will be accrued and treated at the next higher dose level.

7.44 After enrolling 6 patients on a specific dose level, if DLT is observed in at least 2 of 6 patients, then the MTD will have been exceeded and defined as the previous dose unless only 3 patients were treated at the lower dose level. In that case, 3

additional patients will be treated at this lower dose level such that a total of 6 patients are treated at the MTD to more fully assess the toxicities associated with the MTD.

7.45 If one DLT is observed out of 6 patients accrued to dose level 1 on Arm A, we will accrue patients to a middle dose level of $3*10^5$.

7.5 Once the MTD is determined per Section 7.4 for Arm B, an additional 4 patients (for a total of 10) will be treated at the Arm B MTD to further assess toxicity and translational endpoints (see section 14).

7.6 Dose de-escalation: If dose-limiting toxicity is seen 2 or more patients at dose level 1 for either arm, 3 patients will be entered at dose level 0.

7.61 If no DLT is observed, the MTD will have been determined. If accruing to Arm B, additional patients will be enrolled at dose level 0 as per Section 7.44 to better assess toxicity and translational endpoints. If DLT is seen in 1 of 3 patients, up to 3 additional patients will be enrolled to dose level 0 as per Section 7.43. If DLTs are observed in ≥2 of 6 patients treated at dose level 0 in Arm A, no further doses will be evaluated the study will close to accrual.

7.7 If a patient fails to complete the initial course of therapy for reasons other than toxicity, the patient will be regarded as treatment intolerant and an additional patient will be treated at the current dose level; however, all toxicity information will be utilized in the analysis. For these instances, a specific notation will be made for review by the Cancer Center Clinical Research Administrative Subcommittee (CCCRAS).

7.71 Patients will be removed from the study if any of the following occur; a) patient refuses to continue, regardless of reason; b) the principal investigator deems it in the patient's best interest; c) the patient is unable to continue follow-up; d) a major treatment violation occurs; e) the patient experiences DLT, a serious illness, measles or progressive disease; f) the study is terminated.

7.72 If patient develops grade ≥ 3 neurologic toxicity during the follow-up phase that is thought to be possibly, probably or definitely related to the study agent, study accrual will be temporarily held until the data are discussed with the FDA.

7.73 Accrual for this study will cease permanently if: a) any patient experiences a grade 5 treatment-related toxicity; b) study accrual is completed as per section 16.2.

8.0 Dose Modification

No dose modification is allowed. Patients who experience DLT in Arm B after Day 1 treatment will be taken off treatment.

9.0 Ancillary Treatment

9.1 Patients should receive full supportive care while on this study. This includes blood product support, anticonvulsants, perioperative steroids, antibiotic treatment and treatment of other newly diagnosed or concurrent medical conditions. All blood products and concomitant medications such as antidiarrheals, analgesics, anti-emetics received from the first administration of study agent until 28 days post-resection are to be recorded in the medical record.

Acceptable treatment options for nausea/vomiting include:

- ondansetron 4-8 mg po q 8 hours prn or 4 mg IV q 8 hours prn;
- granisetron 1 mg po q 12 hours prn or 10 mcg/kg IV q 12 hours prn;
- prochlorperazine 5-10 mg po q 6-8 hours prn, 25 mg rectally bid prn, or 2.5-5 mg IV by slow infusion (5 mg/min) q 6 hours prn;
- Lorazepam 0.5-2 mg po q 4-6 hours prn;
- Aprepitant 125 mg po day 1, 80 mg po on days 2 and 3;
- Palonosetron 0.25 mg IV (one time dose).

Acceptable treatment options for diarrhea include: loperamide 4 mg po after first diarrheal bowel movement and 2 mg po after each subsequent one up to 16 mg a day and atropine sulfate/diphenoxylate hydrochloride (Lomotil) 2 tab po q 6 hours prn or 10 ml q 6 hours prn.

Acceptable treatment options for fever include acetaminophen 500 mg po 1-2 tabs q 4-6 hours prn up to 4 grams a day.

9.2 Diagnosis of measles in this trial is based on the CDC definition of clinical measles and includes:

- a generalized rash lasting ≥3 days, and
- temperature ≥38.3°C (≥101°F), and
- cough, coryza, and conjunctivitis.

9.3 Since measles virus immunity is required for employment at Mayo, administration of the agent is not expected to result in any risk for nursing or ancillary staff.

9.4 Should a patient develop measles, treatment with intravenous immune globulin will be administered 400 mg/kg/d for 3-5 days. Patients who develop measles will be removed from treatment and followed per observation schedule in Section 4.0 for up to 15 years for evidence of persistent toxicity.

9.5 Patients participating in Mayo Clinic Cancer Center Phase I Program clinical trials are not to be considered for enrollment in any other study involving a pharmacologic agent (drugs, biologics, immunotherapy approaches, gene therapy) whether for symptom control or therapeutic intent.

10.0 Adverse Event (AE) Reporting and Monitoring

10.1 This study will utilize the Common Terminology Criteria for Adverse Events (CTCAE) v3.0 for adverse event monitoring and reporting. The CTC v3.0 can be accessed via the CTEP home page: http://ctep.cancer.gov/. All appropriate treatment areas should have access to a copy of the CTCAE v3.0.

10.11 Adverse event monitoring and reporting is a routine part of every clinical trial. First, identify and grade the severity of the event using the CTCAE. Next, determine whether the event is expected or unexpected (refer to Sections 10.12 and 15.0) and if the adverse event is related to the medical treatment or procedure (see Section 10.13). With this information, determine whether an adverse event should be reported as an expedited report (see Section 10.2) in addition to the routinely reported clinical data (see Sections 10.31 and 18.0).

Expedited adverse event reporting requires submission of a written report, but may also involve telephone notifications. Telephone and written reports are to be completed within the timeframes specified in Section 10.2. All expedited adverse event reports should also be submitted to the local Institutional Review Board (IRB).

10.12 Expected vs. Unexpected

- The determination of whether an AE is expected is based on agent-specific adverse event information provided in Section 15.0 of the protocol.
- Unexpected AEs are those not listed in the agent-specific adverse event information provided in Section 15.0 of the protocol.

10.13 Assessment of Attribution

When assessing whether an adverse event is related to a medical treatment or procedure, the following attribution categories are utilized:

Definite - The adverse event *is clearly related* to the investigational agent(s).
Probable - The adverse event *is likely related* to the investigational agent(s).
Possible - The adverse event *may be related* to the investigational agent(s).
Unlikely - The adverse event *is doubtfully related* to the investigational agent(s).
Unrelated - The adverse event *is clearly NOT related* to the investigational agent(s)

10.2 Expedited Adverse Event Reporting Requirements

Phase I, II and III Studies (Investigational)

	Grade 4 or 5[1] Unexpected with Attribution of Possible, Probable, or Definite	Other Grade 4 or 5 or Any hospitalization during treatment[6]	Secondary AML/MDS[2]
Notify the Cancer Center SAE Coordinator [3] within 24 hours	X		
Submit written report within 5 working days[4, 7]	X		
NCI/CTEP Secondary AML/MDS Report Form within 15 working days[5, 7]			X
Submit Grade 4 or 5 Non-AER Reportable Events/Hospitalization Form within 5 working days.[6, 7]		X[6]	
Submit Serious Adverse Event Reporting Form for Human Gene Transfer Clinical Studies [3, 7, 8]	X		

1. Includes all deaths within 30 days of the last dose of investigational agent regardless of attribution or any death attributed to the agent(s) (possible, probable, or definite) regardless of timeframe.
2. Reporting for this AE required during or after treatment.
3. Notify the Cancer Center SAE Coordinator (P. McNamara, Mayo Clinic - Rochester) by telephone (507) 266-3028 and/or submit a written event summary via fax to (507)284-9628. The Cancer Center SAE Coordinator will subsequently notify the Cancer Center IND Coordinator (see Footnote 7).
4. Use *Adverse Event Expedited Report – Single Agent* or *Multiple Agents* report form. Submit to the Cancer Center SAE Coordinator for further processing (see Footnote 7) and to the Cancer Center CRO Safety inbox for IRB reporting.
5. Submit per form-specified instructions and provide copy to the Cancer Center CRO Safety inbox for IRB reporting and the Cancer Center SAE Coordinator for further processing (see Footnote 7).
6. In addition to standard reporting mechanism for this type of event, submit information to the Cancer Center CRO Safety inbox for IRB reporting and to the Cancer Center SAE Coordinator for further processing (see footnote 7). If Adverse Event Expedited Report – Single Agent or Multiple Agents report form was completed, this form does not need to be completed.
7. The SAE Coordinator will route these reports to the Cancer Center IND Coordinator who, in consultation with the IND holder, will report to the Food and Drug Administration (FDA) as warranted by the event and required by U.S. regulations.
8. This reporting is in addition to all other reporting. Submit form to the Office of Recombinant DNA Activities, NIH, MSC 7010, 600 Executive Boulevard, Suite 302, Bethesda MD 20892-7010; Phone (301) 496-9838. Submit copies of the report to the Cancer Center CRO Safety inbox for IRB reporting and to the Cancer Center SAE Coordinator for further processing (see Footnote 7).

10.3 Adverse events to be graded at each evaluation and pretreatment symptoms/conditions to be evaluated at baseline per Common Terminology Criteria for Adverse Events (CTCAE) v3.0 grading unless otherwise stated in the table below:

Category	Adverse events/Symptoms	Baseline	Each evaluation
Constitutional Symptoms	Fever (in the absence of neutropenia, where neutropenia is defined as ANC <1.0 x 10^9/L)	X	X
	Rigors/Chills	X	X
	Fatigue	X	X
Dermatology/Skin	Rash/desquamation	X	X
Gastrointestinal	# of stools per day	X	
	Diarrhea		X
Pain	Pain – Head/headache	X	X
Neurology	Neuropathy: motor	X	X
	Neuropathy: sensory	X	X
	Ataxia (incoordination)	X	X
	Seizure	X	X
	Speech impairment	X	X
	Confusion	X	X

10.31 Submit via appropriate MCCC Case Report forms (i.e., paper or electronic, as applicable) the following AEs experienced by a patient and not specified in Section 10.3

10.311 Grade 1 and 2 AEs deemed *possibly, probably, or definitely* related to the study treatment or procedure.

10.312 Grade 3 and 4 AEs regardless of attribution to the study treatment or procedure

10.313 Grade 5 AEs (Deaths)

10.3131 Any death within 30 days of the patient’s last study treatment or procedure regardless of attribution to the study treatment or procedure

10.3132 Any death more than 30 days after the patient’s last study treatment or procedure that is felt to be at least possibly treatment related must also be submitted as a Grade 5 AE, with a CTCAE type and attribution assigned.

11.0 Treatment Evaluation

For the purposes of this study, patients should be re-evaluated at 28 days after tumor resection and every 2 months thereafter until progression. If objective response is observed, a confirmatory scan is required at 4 weeks after the objective response.

11.1 Response criteria: The neurologic examination and the MRI and/or CT at each evaluation will be scored as follows:

11.11

NEURO EXAM STATUS (compared to pre-Rx exam)	
Better:	must be on stable or decreasing dose of steroids.
Same:	failure to qualify for better or worse.
Worse:	includes patients requiring increasing steroid dose to remain stable.

11.12 <u>MRI AND/OR CT ASSESSMENT (compared to pretreatment scan for bidimensionally measurable disease):</u>

CR =	total disappearance of all tumor with patient off corticosteroids or only on adrenal replacement maintenance.
PR=	≥50% reduction in product of perpendicular diameters of contrast enhancement or mass with no new lesions with the patient being on stable or decreased steroid dose.
STAB =	failure to qualify for CR, PR, REGR, or PROG.
PROG =	>25% increase in product of perpendicular diameters of contrast enhancement or mass or appearance of new lesions.

11.13 <u>MRI AND/OR CT ASSESSMENT (compared to pretreatment scan) for evaluable disease (i.e., contrast enhancing mass on MRI and/or CT that is not bidimensionally measurable but clearly evaluable for response to therapy</u>.

CR =	total disappearance of all tumor with patient off corticosteroids or only on adrenal replacement maintenance.
REGR =	unequivocal reduction in size of contrast-enhancement or decrease in mass effect as agreed upon independently by primary physician and quality control physicians; no new lesions. Patient should be on stable or decreased steroid dose.
STAB =	failure to qualify for CR, PR, or PROG.
PROG =	unequivocal increase in size of contrast enhancement or increase in mass effect as agreed upon independently by primary physician and quality control physicians: appearance of new lesions.

11.14 Determination of Objective Response Status

<table>
<tr><th rowspan="2">NEURO STATUS</th><th colspan="5">MRI and/or CT Status</th></tr>
<tr><th>CR</th><th>PR</th><th>REGR</th><th>STAB</th><th>PROG</th></tr>
<tr><td>Better</td><td rowspan="2">CR</td><td rowspan="2">PR</td><td rowspan="2">REGR</td><td rowspan="2">STAB</td><td>UNKN*</td></tr>
<tr><td>Same</td><td rowspan="2">PROG</td></tr>
<tr><td>Worse</td><td colspan="4">UNKN*</td></tr>
</table>

*Re-image at next evaluation as per protocol and assess as per table below:

<table>
<tr><th rowspan="2">NEURO STATUS</th><th colspan="5">MRI and/or CT Status</th></tr>
<tr><th>CR</th><th>PR</th><th>REGR</th><th>STAB</th><th>PROG</th></tr>
<tr><td>Better</td><td rowspan="2">CR</td><td rowspan="2">PR</td><td rowspan="2">REGR</td><td rowspan="2">STAB</td><td rowspan="3">PROG</td></tr>
<tr><td>Same</td></tr>
<tr><td>Worse</td><td colspan="4"></td></tr>
</table>

11.15 Survival and Time to Progression Definitions

11.151 Progression-free survival is defined as the length of time from date of registration to a) date of progression or death due to any cause or b) last follow-up.

11.152 Overall survival is defined as the length of time from date of registration to a) death due to any cause or b) last follow-up.

12.0 Descriptive Factor

12.1 Age (years): <40 vs. 40-60 vs. >60.

12.2 Enzyme-inducing anticonvulsant use at study entry: Yes vs. no.

12.3 ECOG PS: 0 vs. 1 vs. 2.

12.4 Prior nitrosoureas: Yes vs. no.

12.5 Interval since end of RT (months).

12.6 Corticosteroid therapy at study entry: Yes vs. no.

12.7 Extent of primary resection: None vs. biopsy vs. subtotal resection vs. gross total resection.

12.8 Extent of resection at recurrence: None vs. biopsy vs. subtotal resection vs. gross total resection.

12.9a Family history of brain tumor: Yes vs. no.
If yes, check all that apply:
_____ Father
_____ Mother
_____ Brother/Sister
_____ Child
_____ Other (list:___________________________________)

12.9b Primary indicator: Measurable disease vs. evaluable disease.

12.9c Number of prior chemotherapy regimens for progressive/recurrent disease.

13.0 Treatment/Follow–up Decision at Evaluation of Patient

13.1 After treatment completion patients will be followed every 2 mos until progression and subsequently as per section 4.0.

13.2 Patients who have been taken off treatment because of unacceptable toxicity will be followed as per observation schedule (Section 4.0).

14.0 Pharmacologic/Correlative

14.1 Tumor Tissue Analysis

14.11 Arm B:
Tissue samples from previous surgery or biopsies will be obtained. CD46 receptor levels will be determined by IHC. The treated tumor will be resected on day 5. H&E stains on the resected tumor and surrounding normal brain will be performed to assess syncytia formation and cytopathic effect and compared with pretreatment tissue sample. If an inflammatory infiltrate is observed it will be further characterized by IHC (CD68, CD45, CD3, CD20 stains). The resected tumor and surrounding normal brain tissue will be examined by IHC for expression of the measles virus N protein and by in situ hybridization and Vero cell overlay for the presence of replicating virus. Baseline pre-treatment tissue will be tested in comparison. Viral gene expression and replication will be characterized in relationship to the distance from the injection site (marked by the catheter). Because measles virus induced cell death is mediated by apoptosis, a TUNEL assay will also be performed using the

Dead End Colorimetric TUNEL System (Promega). Correlative analysis to assess the presence of the virus in tissue and the TUNEL assay will be performed in Dr. Galanis' laboratory using established methodology.

14.12 **Arm A:**
The resected tumor will be examined by H & E. If immune infiltrate is present, it will be characterized by IHC as described in 14.1. CD46 levels will be determined by IHC.

14.2 **Assessment of immune competence**: This will be performed by assessment of immunoglobulin levels, CD4 and CD8 counts, and CD4/CD8 ratio prior to treatment, and at 28 days after tumor resection.

14.3 **Assessment of viremia and viral shedding**. Testing will be performed in Dr. Galanis' laboratory.
Patient's peripheral blood mononuclear cells will be monitored for the presence of measles virus genome. This will be performed by quantitative RT-PCR for the measles virus N mRNA to be performed as follows: a) Arm B: prior to treatment and on days 3, 5, 8. Also on days 14 and 28 after resection and every 2 months thereafter until progression. Subsequent testing as per section 4.0. Testing for viremia will be discontinued after day 28 if two consecutive negative tests are obtained. b) Arm A; prior to treatment, on days 3, 14 and 28 after resection and every 2 months thereafter until progression. Subsequent testing as per section 4.0. Testing for viremia will be discontinued after day 28 if two consecutive negative tests are obtained.

Viral shedding will be assessed by RT-PCR of throat gargle specimen(s) and urine samples as follows: Arm B; baseline, on days 3, 8, also on days 14 and 28 after resection. Arm A; baseline, on days 3, 14 and 28 after tumor resection. Patients will have the gargle and urine specimens done in the CRU as an outpatient visit when patient is not hospitalized. Paula Zollman will be the lab contact, located in Guggenheim 1802, phone 6-0386.

If a patient is found to be shedding the virus in urine or throat gargle specimens(s), family members who don't have documentation of immunity, will be offered testing to assess anti-measles virus immunity by Enzyme Immunoassay (Diamedix, see section 3.18). Measles vaccination will be offered to seronegative individuals, as per standard clinical practice.

14.4 **Assessment of peripheral immune response to viral administration**: measles virus specific immunity will be measured by means of (a) measuring anti-measles virus specific antibodies (IgG) at baseline, at 28 days after tumor resection and every 2 months until progression, and (b) lymphoproliferative assay and IFN-γ ELISPOT performed at baseline and at 28 days after tumor resection. This testing will be performed in Dr. Greg Poland's laboratory.

14.5 **CEA monitoring as in section 4.0**. The assay will be performed at the Mayo Clinic Central Clinical Lab using the Bayer Advia Centaur Immunoassay system (Bayer, Tarrytown, NY).

15.0 Drug Information

15.1 MV-CEA (CEA-Measles Virus – Edmonston Strain)

15.11 Preparation and storage: MV-CEA will be prepared at the Vector Production Core of the Molecular Medicine Program and stored at -80°C. The virus will be thawed and mixed with NS immediately prior to administration.

15.12 Known potential toxicities:

15.121 The most common adverse effect noted is burning and stinging at the site of injection; however, this is not applicable in this study given CNS administration. Occasionally, moderate fever 38.3°-39.4°C have been noted in the month after vaccination, usually within 5-12 days after injection. Rash, which is usually minimal, has been noted. Less commonly, high fever over 39.4°C or mild lymphadenopathy have been reported.

15.122 Occasional reactions:

- Moderate to high fever lasting 1-2 days, starting within a week or two of the vaccination
- A rash, lasting 1-2 days
- Cough and rhinitis
- Erythema multiforme (skin rash)

15.123 Unexpected and rare reactions associated with measles vaccines:

- Allergic reactions to the vaccine including anaphylaxis
- Reactions at the injection site such as wheal, flare or urticaria
- Thrombocytopenia
- Diarrhea

15.13 Nursing guidelines

Due to the very early investigational nature of this drug, no nursing guidelines are known. Please monitor patients closely and follow Section 10.0. More than 90% of the U.S. population has measles virus immunity as a result of natural infection or immunization. Immune status for measles virus is mandatory for Mayo Medical Center personnel. Therefore administration of the agent is not expected to result in any risk for nursing or ancillary staff.

16.0 Statistical Considerations and Methodology

16.1 Study design

This is a single arm, Phase I study designed to determine the MTD and toxicity of attenuated MV-CEA virus in the treatment of recurrent high grade gliomas.

16.11 Sample size: This phase I study may involve a minimum of 21 patients [12 Arm A patients (3 each at dose levels 1 and 2 and 6 at dose level 3) and 9 Arm B patients (3 at level 1 and 6 at level 2)] and a maximum of 36 patients [24 Arm A patients (6 at each of dose levels 1,1.5,2,3) and 12 arm B patients (6 at dose levels 1 and 2)] but would likely require only 24 patients[12 Arm A patients (3 at dose levels 1 and 2 and 6 at 3) and 12 Arm B patients (6 at dose levels 1 and 2)]. An additional 4 patients will be accrued at the Arm B MTD, for a total of 10 patients at the MTD, in order

to further study toxicity and correlative endpoints; thus the study may enroll a maximum of 40 patients with an expected number of 28 patients.

16.12 MTD Determination: MTD is defined as the dose level below the lowest dose that induces dose-limiting toxicity in at least one-third of patients (at least 2 of a maximum of 6 new patients) or the highest dose level for Arm B (2×10^7 TCID50), if ≤ 1 of 6 patients experience DLT. See Section 7.4 for the algorithm on how the MTD will be determined.

16.13 Accrual and Study Duration: Accrual of recurrent high grade glioma patients to recent Mayo/NCCTG neuro-oncology trials (N997B, 987254, 967251) has been about 24 patients per year. In the most recent trial for recurrent GBM patients (N047B), 10 patients have been accrued so far between activation on 9/23/05 and 12/01/2005. These trials have been limited to patients with only one prior regimen for recurrent disease, while the MV-CEA trial does not have such limitation. Only patients who are candidates for gross-total or subtotal tumor resection are eligible, however. Thus, we expect an accrual to this study of about 8-10 patients per year, with completion of accrual in 36-42 months from study initiation.

16.2 Analysis plans: All the relevant results pertaining to toxicity, MTD, response, timed endpoints and laboratory correlates will be examined in an exploratory and hypothesis generating fashion. The small sample size and the heterogeneous patient population associated with phase I studies restricts the generalizability of the results. Any notable statistical result should only be viewed as an impetus for further study in phase II trials rather than a definitive finding in and of itself. All of the following analyses will be performed for Arms A and B.

16.21 Adverse Events Profile
The number and severity of all adverse events (overall, by dose-level, and by tumor group) will be tabulated and summarized in this patient population. The grade 3+ adverse events will also be described and summarized in a similar fashion. This will provide an indication of the level of tolerance of this treatment combination in this patient group.

16.22 Toxicity Profile

As per NCI CTCAE v3.0, the term toxicity is defined as adverse events that are classified as either possibly, probably, or definitely related to study treatment. Non-hematologic toxicities will be evaluated via the ordinal CTC standard toxicity grading. Hematologic toxicity measures of thrombocytopenia, neutropenia, and leukopenia will be assessed using continuous variables as the outcome measures (primarily nadir) as well as categorization via CTC standard toxicity grading.

Overall toxicity incidence as well as toxicity profiles by dose level, patient and tumor site will be explored and summarized. Frequency distributions, graphical techniques and other descriptive measures will form the basis of these analyses.

16.23 Response Profile
Best response is defined to be the best objective status recorded from the start of the treatment until disease progression/recurrence (taking as reference for progressive disease the smallest measurements recorded since the treatment started). The patient's best response assignment will be determined per Section 11.0.

The number of responses is a secondary outcome measure in this trial. Responses will be summarized by simple descriptive summary statistics delineating response type (CR vs PR vs REGR), as well as stable and progressive disease in this patient population.

Percentage of patients who are progression free at 3 and 6 months (PFS3 and PFS6) will be similarly summarized descriptively. Time to disease progression and survival will be reported using standard Kaplan-Meier estimation method.

16.24 Timed Endpoints
The data on time-related variables will be summarized descriptively. These include time until any treatment related toxicity, time until treatment related grade 3+ toxicity, time until hematologic nadirs (WBC, ANC, platelets), time to progression and time to treatment failure, where time to treatment failure is defined as the time from registration to documentation of progression, unacceptable toxicity, or refusal to continue participation by the patient.

16.25 Laboratory Correlates
Descriptive statistics and simple scatterplots will form the basis of presentation of these data. Correlates between these laboratory values and other outcome measures like response and dose levels will be carried out in an exploratory manner. In particular, viremia, CEA titers, viral propagation in tumor, viral shedding, CD46 status, DTH results, CD4 and CD8 counts, LPA and ELISPOT results will be explored in relation to response and toxicity.

16.3 Inclusion of Women and Minorities

This study will be available to all eligible patients, regardless of gender, race or ethnic group. There is no information currently available regarding differential agent effects in subjects defined by race or ethnicity. The planned analyses will, as always, look for differences in treatment effect based on racial groupings. The sample sizes of phase I

studies, however, are not sufficient to provide power for such subset analyses.

To predict the characteristics of patients likely to enroll in this trial we have reviewed the Mayo registration classified by race. This revealed that roughly 3% of patients registered into cancer trials during the past five years could be classified as minorities.

This would suggest that only one or two patients in the study sample are expected to be classified as minorities. This precludes the possibility of a separate subset analysis beyond simple inspection of results for the one or two minority patients.

17.0 Pathology Considerations

Central pathology review is mandatory prior to study entry to confirm eligibility. This will be performed by Dr. Giannini and colleagues

.

18.0 Data Collection Procedure

18.1 Submission Time Table

	≤14 days after registration	Day 14 after resection	Day 21 after resection[4]	Day 28 after resection	OBS[1]	At PROG	Death	At Each Occurrence		
								ADR / AER	New Primary	Late Adverse Event
On-Study Form	X									
Baseline Adverse Events Form	X									
Measurement Form	X	X	X[4]	X	X[1]					
Pathology Reporting Form	X									
Specimen Submission Form	X[5]		X[4]							
Concurrent Treatment Log	X	X	X[4]	X	X[2]					
Nadir/Adverse Event Form		X	X[4]	X	X[1]					
Evaluation/Treatment Form		X[5]	X[4]							
End of Active Treatment Cancel Notification Form		X	X[4]							
Other Laboratory Form	X		X[4]	X	X[3]					
Evaluation/Observation Form				X	X[1]					
Event Monitoring Form						X	X		X	X
Secondary AML/MDS Report Form (see Section 10.0)								X		
ADR/AER (see Section 10.0)								X		

1. see Section 4.0 for observation schedule.
2. Not required after 12 month post-progression evaluation.
3. See Section 4.0 to determine if form necessary.
4. Arm A Cohort 1 Patients only
5. Does not apply to Arm A Cohort 1 Patients (see 4)

19.0 Budget Considerations

19.1 Costs charged to patient: Routine clinical care. The MV-CEA will be provided free of charge by Mayo. However, the participant may be billed for ancillary expenses such as any oral medications prescribed at the time of discharge.

19.2 Tests to be research funded: Research MRIs (or CTs), all pharmacological/correlative

studies, and procedures done solely for study purposes.

20.0 References

Anderson 2004 Anderson BD, Nakamura T, Russell SJ and Peng KW. High CD46 receptor density on tumor cells leads to preferential killing by oncolytic measles virus. Cancer Res 2004, 64:4919-4926, 2004.

Berger M, Prados M, van Gilder J: Phase II trial of GLI328 HSV-tk gene therapy in recurrent glioblastoma. Cancer Gene Ther 4 (suppl):542, 1997

Chiocca EA, Abbed KM, Tatter S, et al: A phase I open-label, dose-escalation, multi-institutional trial of injection with an E1B-Attenuated adenovirus, ONYX-015, into the peritumoral region of recurrent malignant gliomas, in the adjuvant setting. Mol Ther 10:958-966, 2004.

Cutts FT and Markowitz LE. Successes and failures in measles control. The Journal of Infectious Diseases.170 (Suppl 1):S32-41, 1994.

Dorig RE, Marcil A, Chopra A, Richarson CD. The human CD46 molecule is a receptor for measles virus (Edmonston strain). Cell 74:295-305, 1993.

Driesse MJ, Vincent AJ, Sillevis Smitt PA, Kros JM, Hoogerbrugge PM, Avezaat CJ, Valerio D, Bout A.. Intracerebral injection of adenovirus harboring the HSVtk gene combined with ganciclovir administration: toxicity study in nonhuman primates. Gene Ther 5(8):1122-1129, 1998.

Griffin D, Bellini W. Measles Virus in *Fields Virology, e*dited by Fields B, Knipe D, & Howley P, 3:12267-1312, Lippincott-Raven Publishers, 1996.

Jemal A. Cancer statistics, 2005. CA Cancer J Clin 55: 10-30., 2005.

Lang FF, Bruner JM, Fuller GN, Aldape K, Prados MD, Chang S, Berger MS, McDermott MW, Kunwar SM, Junck LR, Chandler W, Zwiebel JA, Kaplan RS, Yung WK. Phase I trial of adenovirus-mediated p53 gene therapy for recurrent glioma: biological and clinical results. J Clin Oncol 21(13):2508-2518, 2003.

Markert JM, Medlock MD, Rabkin SD, et al: Conditionally replicating herpes simplex virus mutant, G207 for the treatment of malignant glioma: results of a phase I trial. Gene Ther 7:867-74., 2000

Mrkic B, Pavlovic J, Rulicke T, Volpe P, Buchholz CJ, Hourcade D, Atkinson JP, Aguzzi A, Cattaneo R. Measles virus spread and pathogenesis in genetically modified mice. J Virol 72:7420-7427, 1998.

Oldfield EH, Ram Z, Culver KW, et al: Gene therapy for the treatment of brain tumors using intra-tumoral transduction with the thymidine kinase gene and intravenous ganciclovir. Hum Gene Ther 4:39-69., 1993

Phuong LK, et al. Use of a vaccine strain of measles virus genetically engineered to produce carcinoembryonic antigen as a novel therapeutic agent against glioblastoma multiforme. Cancer Res 63:2462-9, 2003.

Peng KW, TenEyck C, Galanis E, Kalli K, Hartmann LC, Russell SJ. Intraperitoneal therapy of ovarian cancer using an engineered trackable vaccine strain of measles virus. Cancer Res. 2002 Aug 15;62(16):4656-62.

Peng KW, et al. Non-invasive *in vivo* monitoring of trackable viruses expressing soluble marker peptides. Nat Med 8(5):527-31, 2002.

Rainov NG: A phase III clinical evaluation of herpes simplex virus type 1 thymidine kinase and ganciclovir gene therapy as an adjuvant to surgical resection and radiation in adults with previously untreated glioblastoma multiforme. Hum Gene Ther 11:2389-401., 2000

Ram Z, Culver KW, Oshiro EM, et al: Therapy of malignant brain tumors by intratumoral implantation of retroviral vector-producing cells. Nat Med 3:1354-61., 1997

Rampling R, Cruickshank G, Papanastassiou V, et al: Toxicity evaluation of replication-competent herpes simplex virus (ICP 34.5 null mutant 1716) in patients with recurrent malignant glioma. Gene Ther 7:859-66., 2000

Shand N, Weber F, Mariani L, et al: A phase 1-2 clinical trial of gene therapy for recurrent glioblastoma multiforme by tumor transduction with the herpes simplex thymidine kinase gene followed by ganciclovir. GLI328 European-Canadian Study Group. Hum Gene Ther 10:2325-35., 1999

Shapiro SS, Wilk MB. An analysis of variance test for normality. Biometrics 52:591-610, 1965.

Simon RM, Friedlin B, Rubinstein LV, Arbuck S, Collins J, Christian M. Accelerated titration designs for phase I clinical trials in oncology. JNCI 89:1138-1147, 1997.

Storer B. Design and analysis of phase I trials. Biometrics 45:925-937, 1989.
