# Supplementary material for: Carcinoembryonic antigen-expressing oncolytic measles virus derivative in recurrent glioblastoma: a phase 1 trial
Source: Nat Commun. 2024 Jan 12;15:493. doi: 10.1038/s41467-023-43076-7 (PMC10786937; doi:10.1038/s41467-023-43076-7)
Supplement: Supplementary file 3 — Description of Additional Supplementary Files [file 41467_2023_43076_MOESM3_ESM.pdf]

Description of Additional Supplementary Files

Supplementary Data 1. Gene counts for ISG expression in Group B Patients.

Supplementary Data 2. Immune profiling panel employed for gene expression analysis (Nanostring Technologies).
