# Supplementary material for: Carcinoembryonic antigen-expressing oncolytic measles virus derivative in recurrent glioblastoma: a phase 1 trial
Source: Nat Commun. 2024 Jan 12;15:493. doi: 10.1038/s41467-023-43076-7 (PMC10786937; doi:10.1038/s41467-023-43076-7)
Supplement: Supplementary file 6 — Reporting Summary [file 41467_2023_43076_MOESM6_ESM.pdf]

nature portfolio

Corresponding author(s): Galanis, Evathia

Last updated by author(s): 10/20/2023

Reporting Summary

Nature Portfolio wishes to improve the reproducibility of the work that we publish. This form provides structure for consistency and transparency in reporting. For further information on Nature Portfolio policies, see our Editorial Policies and the Editorial Policy Checklist.

Statistics

For all statistical analyses, confirm that the following items are present in the figure legend, table legend, main text, or Methods section.

n/a	Confirmed	
☐	☒	The exact sample size (*n*) for each experimental group/condition, given as a discrete number and unit of measurement
☐	☒	A statement on whether measurements were taken from distinct samples or whether the same sample was measured repeatedly
☐	☒	The statistical test(s) used AND whether they are one- or two-sided<br>*Only common tests should be described solely by name; describe more complex techniques in the Methods section.*
☐	☒	A description of all covariates tested
☐	☒	A description of any assumptions or corrections, such as tests of normality and adjustment for multiple comparisons
☐	☒	A full description of the statistical parameters including central tendency (e.g. means) or other basic estimates (e.g. regression coefficient) AND variation (e.g. standard deviation) or associated estimates of uncertainty (e.g. confidence intervals)
☐	☒	For null hypothesis testing, the test statistic (e.g. *F*, *t*, *r*) with confidence intervals, effect sizes, degrees of freedom and *P* value noted<br>*Give P values as exact values whenever suitable.*
☒	☐	For Bayesian analysis, information on the choice of priors and Markov chain Monte Carlo settings
☒	☐	For hierarchical and complex designs, identification of the appropriate level for tests and full reporting of outcomes
☐	☒	Estimates of effect sizes (e.g. Cohen's *d*, Pearson's *r*), indicating how they were calculated

Our web collection on statistics for biologists contains articles on many of the points above.

Software and code

Policy information about availability of computer code

Data collection	Clinical trial data was collected using an electronic data capture system developed at Mayo Clinic, INGRES
Data analysis	Nanostring analysis used nCounter Digital Analyzer software.<br>DLDA scores were calculated using a prediction algorithm developed in previously published literature: Kurokawa C, et al. Constitutive Interferon Pathway Activation in Tumors as an Efficacy Determinant Following Oncolytic Virotherapy. J Natl Cancer Inst 110, 1123-1132 (2018).<br>GeneCodis online resource (http://qenecodis.cnb.csic.es/) was used perform gene/protein enrichment analysis of gene expression data.<br>R version 4.0.3 was used for statistical analyses (R Core Team (2020). R: A language and environment for statistical computing. R Foundation for Statistical Computing, Vienna, Austria. URL https://www.R-project.org/). The R packages used for data visualization and analysis were survival (version 3.3-0), stats (version 4.0.3), and ggplot2 (version 3.3.5).

For manuscripts utilizing custom algorithms or software that are central to the research but not yet described in published literature, software must be made available to editors and reviewers. We strongly encourage code deposition in a community repository (e.g. GitHub). See the Nature Portfolio guidelines for submitting code & software for further information.

Data

Policy information about availability of data

All manuscripts must include a data availability statement. This statement should provide the following information, where applicable:

- Accession codes, unique identifiers, or web links for publicly available datasets
- A description of any restrictions on data availability
- For clinical datasets or third party data, please ensure that the statement adheres to our policy

All requests for raw and analyzed data will be reviewed by the Mayo Clinic (MC) Institutional Review Board (IRB). Patient-related data not included in the manuscript were generated as part of a clinical trial and are subject to patient confidentiality. Any data and materials (e.g., tissue samples or imaging data) that can be shared will need approval from the MC IRB and a material transfer agreement in place; this process requires an avarage of six months. All data shared will be de-identified and will be available for one year after access is granted. Any requests for clinical data should be addressed to the corresponding author Evanthia Galanis (galanis.evanthia@mayo.edu). The remaining data are available within the Article, Supplementary Information or Source Data file. Source data are provided with this paper.

Research involving human participants, their data, or biological material

Policy information about studies with human participants or human data. See also policy information about sex, gender (identity/presentation), and sexual orientation and race, ethnicity and racism.

Reporting on sex and gender	This study was made available to all eligible patients regardless of sex or gender. No subgroup analyses were done by gender due to small sample size. Out of 22 evaluable patients, 11 were male and 11 were female.
Reporting on race, ethnicity, or other socially relevant groupings	This study will be available to all eligible patients, regardless of gender, race or ethnic group. There is no information currently available regarding differential agent effects in subjects defined by race or ethnicity. The planned analyses will, as always, look for differences in treatment effect based on racial groupings. To predict the characteristics of patients likely to enroll in this trial we have reviewed the Mayo registration classified by race. This revealed that roughly 3% of patients registered into cancer trials during the past five years could be classified as minorities. This would suggest that only one or two patients in the study sample are expected to be classified as minorities. This precludes the possibility of a separate subset analysis beyond simple inspection of results for the one or two minority patients.
Population characteristics	The study population is adults 18 and older with recurrent glioblastoma.
Recruitment	Study candidates were identified by treating physicians at Mayo Clinic in Rochester, MN.
Ethics oversight	Mayo Clinic (MC) Institutional Review Board (IRB) provided ethics oversight for this trial.

Note that full information on the approval of the study protocol must also be provided in the manuscript.

Field-specific reporting

Please select the one below that is the best fit for your research. If you are not sure, read the appropriate sections before making your selection.

☒ Life sciences ☐ Behavioural & social sciences ☐ Ecological, evolutionary & environmental sciences

For a reference copy of the document with all sections, see nature.com/documents/nr-reporting-summary-flat.pdf

Life sciences study design

All studies must disclose on these points even when the disclosure is negative.

Sample size	This phase I/II trial enrolled patients to two treatment regimens in a standard 3+3 cohort design, with additional patients enrolled in a maximum tolerated dose (MTD) expansion cohort. Accrual in Group A occurred until the MTD of MV-CEA was determined following single dose administration. Subsequently, patients were enrolled in Group B for further evaluation of the MTD and to further study toxicity and correlative endpoints. In total, 23 patients were enrolled: 10 patients (9 evaluable) in Group A and 13 patients in Group B. Patients were assigned to Group A or Group B sequentially; these study arms were not open to accrual simultaneously.
Data exclusions	As was pre-specified in the study protocol, evaluable patients were those who gave their informed consent and received MV-CEA treatment. One patient in Group A was excluded due to not receiving study treatment.
Replication	Despite the fact that all patients were immune to the virus per study design and FDA mandate in order to increase safety, systemic pre-existing immunity did not block replication in the tumor, following intratumoral administration.
Randomization	The primary goal of this study was determination of maximum tolerated dose of study drug and therefore patients were not randomized.
Blinding	This phase I/II study had a non-randomized design and was focused on safety thus blinding was not used.

Reporting for specific materials, systems and methods

We require information from authors about some types of materials, experimental systems and methods used in many studies. Here, indicate whether each material, system or method listed is relevant to your study. If you are not sure if a list item applies to your research, read the appropriate section before selecting a response.

Materials & experimental systems

n/a	Involved in the study
☐	☒ Antibodies
☒	☐ Eukaryotic cell lines
☒	☐ Palaeontology and archaeology
☒	☐ Animals and other organisms
☐	☒ Clinical data
☒	☐ Dual use research of concern
☐	☐ Plants

Methods

n/a	Involved in the study
☒	☐ ChIP-seq
☒	☐ Flow cytometry
☒	☐ MRI-based neuroimaging

Antibodies

Antibodies used	Immunohistochemical stains were performed utilizing antibodies directed against CD3 (clone LN10, Leica Biosystems, United Kingdom), CD4 (clone SP35, Ventana, USA), CD8 (clone C8, Dako, Denmark), CD20 (clone L26, Dako, Denmark), and CD68 (clone KP1, Dako, Denmark).
Validation	Validation provided by the manufacturer in addition to clinical validation by the performing laboratory.

Clinical data

Policy information about clinical studies

All manuscripts should comply with the ICMJEguidelines for publication of clinical research and a completedCONSORT checklist must be included with all submissions.

Clinical trial registration	NCT00390299
Study protocol	Data was collected during clinical assessments made from 2006 to 2018. All assessments were made at the Mayo Clinic in Rochester, MN with the exceptions of assessments made during the post-treatment observation period which could take place at patients' local physician provided no evidence of viremia. In these cases follow-up imaging was provided to study team at Mayo Clinic in Rochester, MN. Protocol supplied as supplementary note.
Data collection	Primary outcomes:<br>1. Maximum tolerated dose (MTD) as measured by the number of patients with dose limiting toxicities (DLTs)<br>2. Number of patients experiencing Grade 3+ Adverse Events, per NCI CTCAE version 3.0<br><br>Secondary outcomes:<br>1. Best response, defined as the best objective status recorded from start of treatment until disease progression<br>2. Progression-free survival, defined as length of time from date of registration until date of progression, death due to any cause, or last follow-up<br>3. Survival, defined as length of time from registration until death due to any cause or last follow-up<br><br>Laboratory correlative outcomes include viremia, CEA titers, viral propagation in tumor, viral shedding, and immune filtration in tumor
Outcomes	In this first in human study in glioblastoma the Edmonston measles oncolytic platform demonstrated safety, ability to replicate in the tumor and resulted in promising survival outcomes.
